# Supplementary material for: Parental satisfaction towards care given at neonatal intensive care unit in Ethiopia: A systematic review and meta-analysis
Source: PLoS One. 2024 Dec 5;19(12):e0313451. doi: 10.1371/journal.pone.0313451 (PMC11620403; doi:10.1371/journal.pone.0313451)
Supplement: S2 Table — (DOCX) [file pone.0313451.s002.docx]

**S2 Table. Extracted studies which are eligible for systematic review and meta-analysis of parental satisfaction in Ethiopia.**

| Authors | Study Year | Study area | Study design | Sample Size | Case | Prevalence % | (95% CI) LCI | (95% CI) UCI | Response rate % | Quality score based on NOS | Name of data extractor | Date of data extraction |
| --- | --- | --- | --- | --- | --- | --- | --- | --- | --- | --- | --- | --- |
| Fikadu L. et al. | 2020 | Oromia | Mixed study | 109 | 79 | 72.75 | NA | NA | 100 | 8 | TGH | 02/01/2024 |
| Jamie AH et al. | 2023 | Harar | cross-sectional | 288 | 165 | 57 | NA | NA | 96.3 | 9 | TGH | 02/01/2024 |
| Berhan Y. | 2020 | Addis Ababa | cross-sectional | 286 | 128 | 42.8 | NA | NA | 95.7 | 9 | TGH | 04/01/2024 |
| Ali MS. et al. | 2020 | Amhara | cross-sectional | 300 | 150 | 50 | NA | NA | 94.6 | 8 | TGH | 05/01/2024 |
| Alle YF et al. | 2022 | Amhara | cross-sectional | 385 | 184 | 47.8 | 43.1 | 52.5 | 95.06 | 9 | TM | 02/01/2024 |
| Alemu A. et al. | 2021 | Amhara | cross-sectional | 400 | 214 | 55 | 50 | 59.9 | 97.3 | 9 | TM | 03/01/2024 |
| Endale H. | 2017 | Addis Ababa | cross-sectional | 422 | 167 | 41.8 | NA | NA | 94.7 | 8 | TM | 03/01/2024 |
| Sileshi E et al. | 2022 | SNNPR | cross-sectional | 401 | 240 | 63 | 58 | 68 | 95 | 8 | TM | 02/01/2024 |
| Adal Z et al. | 2021 | Oromia | cross-sectional | 122 | 66 | 57.9 | 49.10 | 66.70 | 93.5 | 9 | AB | 04/01/2024 |
| Mekonnen WN et al. | 2017 | Amhara | cross-sectional | 127 | 98 | 77 | NA | NA | 98.4 | 7 | AB | 04/01/2024 |
| Workie M et al. | 2023 | Harar | cross-sectional | 408 | 206 | 50.5 | 45.6 | 55.5 | 97.6 | 7 | AB | 05/01/2024 |

NOS: Newcastle Ottawa Scale

NA: Not Applicable

SNNPR: Southern Nations, Nationalities, and Peoples' Region

TGH: Teklehaimanot Gereziher Haile

TM: Teklewoini Mariye

AB: Abrha Hailay
