# Supplementary material for: Parental satisfaction towards care given at neonatal intensive care unit in Ethiopia: A systematic review and meta-analysis
Source: PLoS One. 2024 Dec 5;19(12):e0313451. doi: 10.1371/journal.pone.0313451 (PMC11620403; doi:10.1371/journal.pone.0313451)
Supplement: S3 Table — (DOCX) [file pone.0313451.s003.docx]

**S3 Table. Extracted studies for systematic review and meta-analysis of factors associated with parental satisfaction in Ethiopia.**

| Factors | Authors | Study Year | Study area | Study design | Sample Size | No Exposed over total (%) | Exposed over total | Prevalence % | AOR | (95% CI) LCI | (95% CI) UCI | Name of data extractor | Date of data extraction |
| --- | --- | --- | --- | --- | --- | --- | --- | --- | --- | --- | --- | --- | --- |
| Place of Residence | Fikadu L. et al. | 2020 | Oromo | MS | 109 | 32 | 77 | 70.6 | 3.68 | 1.15 | 11.76 | NA | 30/01/2024 |
|  | Jamie AH et al. | 2023 | Harar | CS | 288 | 116 | 172 | 59.72 | 6.94 | 3.42 | 8.06 | NA | 03/02/2024 |
|  | Berhan Y. | 2020 | Addis Ababa | CS | 286 | 62 | 95 | 74.2 | 1.96 | 1.092 | 3.526 | NA | 03/02/2024 |
|  | Alle YF et al. | 2022 | Amhara | CS | 385 | 82 | 116 | 58.6 | 2.94 | 1.42 | 6.06 | NA | 04/02/2024 |
|  | Workie M et al. | 2023 | Harar | CS | 408 | 202 | 206 | 50.5 | 2.13 | 1.33 | 3.43 | GT | 02/02/2024 |
| Birth Weight | Jamie AH et al. | 2023 | Harar | CS | 288 | 115 | 173 | 60.07 | 1.14 | 1 | 3.94 | AB | 04/02/2024 |
|  | Alle YF et al. | 2022 | Amhara | CS | 385 | 66 | 96 | 59.3 | 2.14 | 1.16 | 3.94 | TGH | 04/02/2024 |
| Length of Hospital Stays | Ali MS. et al. | 2020 | Amhara | CS | 300 | 210 | 90 | 42.86 | 2.42 | 1.39 | 4.18 | GT | 05/02/2024 |
|  | Alle YF et al. | 2022 | Amhara | CS | 385 | 104 | 140 | 57.4 | 2.18 | 1.13 | 4.2 | GT | 06/02/2024 |
|  | Alemu A. et al. | 2021 | Amhara | CS | 400 | 124 | 196 | 61.25 | 5.09 | 2.15 | 12.01 | TM | 03/02/2024 |
|  | Workie M et al. | 2023 | Harar | CS | 408 | 202 | 206 | 50.5 | 4.25 | 2.08 | 8.69 | TM | 03/02/2024 |
| Information using direction indicator | Sileshi E et al. | 2022 | SNNPR | CS | 401 | 41 | 163 | 79.9 | 3.14 | 1.85 | 5.31 | TGH | 07/02/2024 |
|  | Mekonnen WN et al. | 2017 | Amhara | CS | 127 | 70 | 57 | 44.9 | 5.16 | 1.51 | 17.6 | GT | 06/02/2024 |

MS: Mixed Study

CS: Cross-Sectional

SNNPR: Southern Nations, Nationalities, and Peoples' Region

NA: Negasi Asres

GT: Girmay Teklay

AB: Abrha Hailay

TGH: Teklehaimanot Gereziher Haile

TM: Teklewoini Mariye
