# Supplementary material for: Parental satisfaction towards care given at neonatal intensive care unit in Ethiopia: A systematic review and meta-analysis
Source: PLoS One. 2024 Dec 5;19(12):e0313451. doi: 10.1371/journal.pone.0313451 (PMC11620403; doi:10.1371/journal.pone.0313451)
Supplement: S4 Table — (DOCX) [file pone.0313451.s004.docx]

**S4 Table.** **Newcastle-Ottawa Quality Assessment Scale.**

| **Authors** | **Selection** | | | | **Comparability** | **Outcome** | | **Total score** |
| --- | --- | --- | --- | --- | --- | --- | --- | --- |
|  | Representativeness s (1) | Sample size (1) | Non respondents (1) | Ascertainment of the exposure (risk factor) (2) | The subjects in different outcome groups are comparable, based on the study design or analysis. confounding factors are controlled (2) | Assessment of the outcome (2) | Statistical test (1) |  |
| Fikadu L. et al. [34] | 1 | 1 | 1 | 2 | 1 | 1 | 1 | 8 |
| Jamie AH et al. [37] | 1 | 1 | 1 | 2 | 1 | 2 | 1 | 9 |
| Workie M et al. [38] | 1 | 1 | 1 | 2 | 1 | 2 | 1 | 9 |
| Berhan Y. [36] | 1 | 1 | 1 | 1 | 1 | 2 | 1 | 8 |
| Ali MS. et al. [31] | 1 | 1 | 1 | 2 | 2 | 1 | 1 | 9 |
| Alle YF et al. [32] | 1 | 1 | 1 | 1 | 2 | 2 | 1 | 9 |
| Alemu A. et al. [33] | 1 | 1 | 1 | 1 | 1 | 2 | 1 | 8 |
| Endale H. [30] | 1 | 1 | 1 | 1 | 2 | 1 | 1 | 8 |
| Sileshi E et al. [39] | 1 | 1 | 1 | 2 | 2 | 1 | 1 | 9 |
| Adal Z et al. [35] | 1 | 1 | 1 | 1 | 1 | 1 | 1 | 7 |
| Mekonnen WN et al. [29] | 1 | 1 | 1 | 1 | 1 | 1 | 1 | 7 |
