# Supplementary material for: Parental satisfaction towards care given at neonatal intensive care unit in Ethiopia: A systematic review and meta-analysis
Source: PLoS One. 2024 Dec 5;19(12):e0313451. doi: 10.1371/journal.pone.0313451 (PMC11620403; doi:10.1371/journal.pone.0313451)
Supplement: S6 Table — (DOCX) [file pone.0313451.s006.docx]

**S6 Table. GRADE assessment to assess the certainty of evidence of this systematic review and meta-analysis of parental satisfaction in Ethiopia.**

| **Domain** | **Definition and Elements** | **Score and Application** |
| --- | --- | --- |
| Study Limitations | Study limitations is the degree to which the included studies for a given outcome have a high likelihood of adequate protection against bias (i.e., good internal validity), assessed through two main elements:   - Study design: Whether RCTs or other designs such as nonexperimental or observational studies. - Study conduct. Aggregation of ratings of risk of bias of the individual studies under consideration. | **Medium** |
| Directness | Directness relates to (a) whether evidence links interventions directly to a health outcome of specific importance for the review, and (b) for comparative studies, whether the comparisons are based on head-to-head studies. The EPC should specify the comparison and outcome for which the SOE grade applies. Evidence may be indirect in several situations such as:   - The outcome being graded is considered intermediate (such as laboratory tests) in a review that is focused on clinical health outcomes (such as morbidity, mortality). - Data do not come from head-to-head comparisons but rather from two or more bodies of evidence to compare interventions A and B—e.g., studies of A vs. placebo and B vs. placebo, or studies of A vs. C and B vs. C but not direct comparisons of A vs. B. - Data are available only for proxy respondents (e.g., obtained from family members or nurses) instead of directly from patients for situations in which patients are capable of self-reporting and self-report is more reliable.   Indirectness always implies that more than one body of evidence is required to link interventions to the most important health outcome. | **Direct** |
| Consistency | Consistency is the degree to which included studies find either the same direction or similar magnitude of effect. EPCs can assess this through two main elements:   - Direction of effect: Effect sizes have the same sign (that is, are on the same side of no effect or a minimally important difference [MID]) - Magnitude of effect: The range of effect sizes is similar. EPCs may consider the overlap of CIs when making this evaluation.   The importance of direction vs. magnitude of effect will depend on the key question and EPC judgments. | **Consistent** |
| Precision | Precision is the degree of certainty surrounding an effect estimate with respect to a given outcome, based on the sufficiency of sample size and number of events.   - A body of evidence will generally be imprecise if the optimal information size (OIS) is not met. OIS refers to the minimum number of patients (and events when assessing dichotomous outcomes) needed for an evidence base to be considered adequately powered. - If EPCs performed a meta-analysis, then EPCs may also consider whether the CI crossed a threshold for an MID. - If a meta-analysis is infeasible or inappropriate, EPCs may consider the narrowness of the range of CIs or the significance level of p-values in the individual studies in the evidence base. | **Precise** |
| Reporting Bias | Reporting bias results from selectively publishing or reporting research findings based on the favorability of direction or magnitude of effect. It includes:   - Study publication bias, i.e., nonreporting of the full study. - Selective outcome reporting bias, i.e., nonreporting (or incomplete reporting) of planned outcomes or reporting of unplanned outcomes. - Selective analysis reporting bias, i.e., reporting of one or more favorable analyses for a given outcome while not reporting other, less favorable analyses.   Assessment of reporting bias for individual studies depends on many factors–e.g. availability of study protocols, unpublished study documents, and patient-level data. Detecting such bias is likely with access to all relevant documentation and data pertaining to a journal publication, but such access is rarely available.  Because methods to detect reporting bias in observational studies are less certain, this guidance does not require EPCs to assess it for such studies. | **Suspected** |

**Note:** CI = confidence internal; EPC = Evidence-based Practice Center; MID = minimally important difference; OIS = optimal information size
