# Supplementary material for: Parental satisfaction towards care given at neonatal intensive care unit in Ethiopia: A systematic review and meta-analysis
Source: PLoS One. 2024 Dec 5;19(12):e0313451. doi: 10.1371/journal.pone.0313451 (PMC11620403; doi:10.1371/journal.pone.0313451)
Supplement: S7 Table — (DOCX) [file pone.0313451.s007.docx]

**S7 Table. Studies identified during the literature search for systematic review and meta-analysis of parental satisfaction in Ethiopia.**

| **S.No.** | **Authors** | **Eligibility** | **Reason for exclusion** |
| --- | --- | --- | --- |
|  | Gulo B. et al. | Excluded from analysis | Duplicate articles |
|  | Mohammed A, Legesse H, Hailu M. |  |  |
|  | Adal Z et al. |  |  |
|  | Mukeshimana E. et al. |  |  |
|  | Alle YF et al. |  |  |
|  | Fikadu L. et al. |  |  |
|  | Jamie AH, Gebremedhin ES. |  |  |
|  | Workie M et al. |  |  |
|  | Ali MS. et al. |  |  |
|  | Mekonnen WN et al. |  |  |
|  | Berhan Y. |  |  |
|  | Alemu A. et al. |  |  |
|  | Endale H. |  |  |
|  | Sileshi E et al. |  |  |
|  | Lumumba PN et al. |  |  |
|  | Butt ML et al. |  |  |
|  | Dall'Oglio I. et al. |  |  |
|  | Bastani F. et al. |  |  |
|  | Yılmaz F et al. |  |  |
|  | Tsironi S et al. |  |  |
|  | Lanlehin R. et al. |  |  |
|  | Voulgaridou A. et al. |  |  |
|  | Srivastava R. et al. |  |  |
|  | Jayasinghe C. et al. |  |  |
|  | McCormick MC et al. |  |  |
|  | Lake ET et al. |  |  |
|  | Conner JM et al. |  |  |
|  | Browne JV et al. |  |  |
|  | Sankar V. et al. |  |  |
|  | Hagen IH et al. |  |  |
|  | Campus MN et al. |  |  |
|  | Nguyen AT et al. |  |  |
|  | Salehi Z. et al. |  |  |
|  | Mitchell-DiCenso A. et al. |  |  |
|  | Latour JM et al. |  |  |
|  | Lilo EA et al. |  |  |
|  | Demis A. et al. |  |  |
|  | Silesh M. et al. |  |  |
|  | Yifru Berhan YB et al. |  |  |
|  | Kidane A. et al. |  |  |
|  | Oude Maatman SM et al. |  |  |
|  | Latour JM, Hazelzet JA et al. |  |  |
|  | Tiryaki O. et al. |  |  |
|  | Ullsten A. et al. |  |  |
|  | Ferentzi H. et al. |  |  |
|  | Ding X. et al. |  |  |
|  | Pereira N. et al. |  |  |
|  | Klein V. et al. |  |  |
|  | Ferreira A. et al. |  |  |
|  | Rennick JE et al. |  |  |
|  | Baldoni F. et al. |  |  |
|  | Terp K. et al. |  |  |
|  | Grandjean C. et al. |  |  |
|  | Cruz AC et al. |  |  |
|  | Marini MM et al. |  |  |
|  | Chen, MT. et al. |  |  |
|  | Matalon, C. et al. |  |  |
|  | Nikolaou, D. |  |  |
|  | James, D. E. et al. |  |  |
|  | Savanheimo N. et al. |  |  |
|  | Yoo, J. et al. |  |  |
|  | Çamur Z. et al. |  |  |
|  | Shah PV et al. |  |  |
|  | Spahr CD et al. |  |  |
|  | Zanobini M. et al. |  |  |
|  | Goetting A. et al. |  |  |
|  | Johnston C. et al. |  |  |
|  | Charbonneau E. et al. |  |  |
|  | Friedman BA et al. |  |  |
|  | Sloper P. et al. |  |  |
|  | Shao M. et al. |  |  |
|  | Tilbury C. et al. |  |  |
|  | Guidubaldi J. et al. |  |  |
|  | Byczkowski TL et al. |  |  |
|  | Fantuzzo J. et al. |  |  |
|  | Warde CM et al. |  |  |
|  | Haines C. et al. |  |  |
|  | Shafer JS et al. |  |  |
|  | Rey JM et al. |  |  |
|  | Summers JA et al. |  |  |
|  | Ygge BM et al. |  |  |
|  | Williams G. et al. |  |  |
|  | Hart CN et al. |  |  |
|  | Pelchat D. et al. |  |  |
|  | Schaffer P. et al. |  |  |
|  | Acs G. et al. |  |  |
|  | Kruszecka-Krówka A. et al. |  |  |
|  | Gibbons S. et al. |  |  |
|  | Mbuvi John B. |  |  |
|  | Galanis P. et al. |  |  |
|  | Segers E. et al. |  |  |
|  | Nieves H. et al. |  |  |
|  | Sakonidou S. et al. |  |  |
|  | Martin AE et al. |  |  |
|  | Muwanga E. |  |  |
|  | Wilunda C. et al. |  |  |
|  | Klemming S. et al. |  |  |
|  | Wondie WT et al. |  |  |
|  | Madara CW. |  |  |
|  | NDAGIJIMANA PC. |  |  |
|  | Birtukan A. |  |  |
|  | Ketema DB |  |  |
|  | Akbar S. |  |  |
|  | Workneh R. |  |  |
|  | Gabra MI et al. |  |  |
|  | Tuglo LS et al. |  |  |
|  | Thommesen TG. |  |  |
|  | Feleke T. |  |  |
|  | Shibiru S. et al. |  |  |
|  | BHAMBRHO BA et al. |  |  |
|  | Sereshti M. et al. |  |  |
|  | Negasi KB et al. |  |  |
|  | Phiri PG, Chan CW, Wong CL. |  |  |
|  | Nigussie T, Azanaw G, Shumye M. |  |  |
|  | Befekadu L. |  |  |
|  | Mulu GB et al. |  |  |
|  | HASHI MH, Farah AM, Ibrahim SJ. |  |  |
|  | Bazambanza D. |  |  |
|  | Betemariem D. et al. |  |  |
|  | Muktar SA et al. |  |  |
|  | Adane H. |  |  |
|  | Forbes F. et al. |  |  |
|  | Jisso M. et al. |  |  |
|  | Masenya SL. |  |  |
|  | Bedane B. et al. |  |  |
|  | Bayou NB et al. |  |  |
|  | Wassihun T. |  |  |
|  | Manongi R. et al. |  |  |
|  | Hana W. |  |  |
|  | Seniwati T, Wanda D, Nurhaeni N. |  |  |
|  | Bekele B. |  |  |
|  | Aarsland M. |  |  |
|  | Tezera N. et al. |  |  |
|  | Gad A. et al. |  |  |
|  | Singh M. et al. |  |  |
|  | Jonsson M. et al. |  |  |
|  | Bonner O. et al. |  |  |
|  | Gehl MB et al. |  |  |
|  | Cricco-Lizza R. |  |  |
|  | McPherson ML et al. |  |  |
|  | Alsalem N. et al. |  |  |
|  | Mercado K, Vittner D, McGrath J. |  |  |
|  | Clark OE et al. |  |  |
|  | Parry T. et al. |  |  |
|  | Coughlin K. et al. |  |  |
|  | Smith JR, Pineda RG. |  |  |
|  | McCarley RM et al. |  |  |
|  | Manalu LO, Sutisna MI. |  |  |
|  | Coffman S, Vitt MJ, Deets C. |  |  |
|  | Damrongrak P. et al. |  |  |
|  | Mimani Minuta W. et al. |  |  |
|  | Bayisenge M. |  |  |
|  | Yismaw AE, Gelagay AA, Sisay MM. |  |  |
|  | Menalu MM et al. |  |  |
|  | Jebessa S. et al. |  |  |
|  | Araya ES. |  |  |
|  | Amuam DK. |  |  |
|  | Dagnaw FT et al. |  |  |
|  | Berihu G. et al. |  |  |
|  | Jabraeili M. et al. |  |  |
|  | Mengistu BA et al. |  |  |
|  | Delele TG et al. |  |  |
|  | Ahmed Mohammed Sabry F et al. |  |  |
|  | dos Santos Oliveira SJ et al. |  |  |
|  | Ibrahim AM et al. |  |  |
|  | Teklegiorgis H, Ketema H. |  |  |
|  | Sánchez-Veracruz MJ et al. |  |  |
|  | Cleveland LM. et al. |  |  |
|  | De Bernardo G. et al. |  |  |
|  | Russell G. et al. |  |  |
|  | Abuqamar M. et al. |  |  |
|  | Ebrahim S. et al. |  |  |
|  | Stevens DC et al. |  |  |
|  | Adama EA et al. |  |  |
|  | Mol C. et al. |  |  |
|  | Heidari H. et al. |  |  |
|  | Jannes C. et al. |  |  |
|  | Voos KC et al. |  |  |
|  | Shrestha T, Bista AP, Shrestha S. |  |  |
|  | Obeidat HM et al. |  |  |
|  | Kadivar M. et al. |  |  |
|  | Cintra CD et al. |  |  |
|  | Liu TT et al. |  |  |
|  | Zorro C. et al. |  |  |
|  | Ding X. et al. |  |  |
|  | Pick V. et al. |  |  |
|  | Dhingra P. |  |  |
|  | Fazio SB et al. |  |  |
|  | Shahheidari M. et al. |  |  |
|  | Mengesha EW et al. |  |  |
|  | Reis MD et al. |  |  |
|  | Orfali K. et al. |  |  |
|  | Abuidhail J. et al. |  |  |
|  | Koontz VS et al. |  |  |
|  | Williams KG et al. |  |  |
|  | Weiss S. et al. |  |  |
|  | Moore KA et al. |  |  |
|  | Ladani MT et al. |  |  |
|  | Gallagher K. et al. |  |  |
|  | Kasat K. et al. |  |  |
|  | Finlayson K. et al. |  |  |
|  | Cescutti‐Butler L. et al. |  |  |
|  | Brødsgaard A. et al. |  |  |
|  | Rihan SH et al. |  |  |
|  | Saxton SN et al. |  |  |
|  | Epstein EG et al. |  |  |
|  | Ghadery-Sefat A. et al. |  |  |
|  | Meert KL et al. |  |  |
|  | Ranchod TM et al. |  |  |
|  | Sikorova L. et al. |  |  |
|  | Franck LS et al. |  |  |
|  | Kjellsdotter A. et al. |  |  |
|  | Zhang R et al. |  |  |
|  | Fotiou C. et al. |  |  |
|  | Smith VC et al. |  |  |
|  | Ramezani T. et al. |  |  |
|  | Holditch-Davis D. et al. |  |  |
|  | Abdel-Latif ME et al. |  |  |
|  | Heinemann AB et al. |  |  |
|  | Nazari R. et al. |  |  |
|  | Oliveira AR et al. |  |  |
|  | Skene C. et al. |  |  |
|  | Zauche LH et al. |  |  |
|  | Wigert H. et al. |  |  |
|  | Baylis R. et al. |  |  |
|  | Lantz B. et al. |  |  |
|  | Serlachius A. et al. |  |  |
|  | Rosenthal SA et al. |  |  |
|  | Tran C. et al. |  |  |
|  | Koliouli F, Gaudron CZ. |  |  |
|  | Balbino FS et al. |  |  |
|  | Raiskila S. et al. |  |  |
|  | Tilaye M. |  |  |
|  | Horwood C. et al. |  |  |
|  | Mariano K. et al. |  |  |
|  | Elshanti A. et al. |  |  |
|  | Abie B, Shehibo A, Terefe B. |  |  |
|  | Haile MT. |  |  |
|  | Mbwele B. et al. |  |  |
|  | Badi MB et al. |  |  |
|  | Siva N. et al. |  |  |
|  | Feleke H. et al. |  |  |
|  | Ahmed M. |  |  |
|  | Lulseged S, Deste C. |  |  |
|  | Bayih WA et al. |  |  |
|  | Ayalew K. |  |  |
|  | Heidarzadeh M. et al. |  |  |
|  | Hewedy AA et al. |  |  |
|  | Talus E. et al. |  |  |
|  | Ismail MS, Mohmmed RG. |  |  |
|  | Enjamo M. et al. |  |  |
|  | Kebede AA et al. |  |  |
|  | Permanasari I. et al. |  |  |
|  | Guye AH et al. |  |  |
|  | Ndwiga C. et al. |  |  |
|  | Abdalsemia Elewa A et al. |  |  |
|  | Duresa WB et al. |  |  |
|  | Gonfa DN et al. |  |  |
|  | Endehabtu BF et al. |  |  |
|  | Berhe ET et al. |  |  |
|  | Lulseged S, D'Este C. |  |  |
|  | Billah SM et al. |  |  |
|  | Heen EK et al. |  |  |
|  | Wycliffe S. |  |  |
|  | Mekuria Y, St Denis C. |  |  |
|  | Mahon P. et al. |  |  |
|  | Reid T. et al. |  |  |
|  | Hurst I. et al. |  |  |
|  | Saunders RP et al. |  |  |
|  | Carter BS et al. |  |  |
|  | Hallowell SG et al. |  |  |
|  | Steedman WK. |  |  |
|  | Doede M. et al. |  |  |
|  | Lin M. et al. |  |  |
|  | Clarke-Pounder JP et al. |  |  |
|  | Marçola L. et al. |  |  |
|  | Maram Abuqamar RN et al. |  |  |
|  | Wong SE et al. |  |  |
|  | Auslander GK et al. |  |  |
|  | Labrie NH et al. |  |  |
|  | Awindaogo F. et al. |  |  |
|  | Misgen M. et al. |  |  |
|  | Govindaswamy P. et al. |  |  |
|  | Dhingra P. et al. |  |  |
|  | Magliyah AF et al. |  |  |
|  | Gavey J. et al. |  |  |
|  | Petteys AR et al. |  |  |
|  | Van Riper M. |  |  |
|  | Mohtashami M. et al. |  |  |
|  | Hames JL et al. |  |  |
|  | Hoge MK et al. |  |  |
|  | Albayrak S. et al. |  |  |
|  | Manning AN. |  |  |
|  | Joseph AM. |  |  |
|  | Gadepalli SK et al. |  |  |
|  | Cheldelin LV et al. |  |  |
|  | Feeley N. et al. |  |  |
|  | Ottosson C. et al. |  |  |
|  | Bruns DA et al. |  |  |
|  | Liu LX et al. |  |  |
|  | Kusnatalia K. et al. |  |  |
|  | Riskin A. et al. |  |  |
|  | Minckas N. et al. |  |  |
|  | Schuler R. et al. |  |  |
|  | Neu M, Klawetter S. et al. |  |  |
|  | Globus O. et al. |  |  |
|  | Ionio C. et al. |  |  |
|  | Thébaud V. et al. |  |  |
|  | Wang L. et al. |  |  |
|  | Toivonen M. et al. |  |  |
|  | Eskandari S. et al. |  |  |
|  | Vazquez V. et al. |  |  |
|  | Jiang S. et al. |  |  |
|  | Matricardi S. et al. |  |  |
|  | Koontz VS. |  |  |
|  | Bertoncelli N. et al. |  |  |
|  | Hall SL et al. |  |  |
|  | Watson J. et al. |  |  |
|  | Carty CL et al. |  |  |
|  | Ribeiro C. et al. |  |  |
|  | Mazlan R. et al. |  |  |
|  | Weis J. et al. |  |  |
|  | Schoenherr J. et al. |  |  |
|  | Judy Levick MS et al. |  |  |
|  | Umberger E. et al. |  |  |
|  | Batton B. et al. |  |  |
|  | Mäkelä H. et al. |  |  |
|  | Swanson JR et al. |  |  |
|  | Enlow E. et al. |  |  |
|  | Franck LS, Oulton K. et al. |  |  |
|  | Beal JA et al. |  |  |
|  | Treherne SC et al. |  |  |
|  | Cooper LG et al. |  |  |
|  | Kenner C. |  |  |
|  | Tiryaki Ö. et al. |  |  |
|  | Kaur M. et al. |  |  |
|  | Schappin R. et al. |  |  |
|  | Lean RE et al. |  |  |
|  | Makkar A. et al. |  |  |
|  | Davidson JE et al. |  |  |
|  | Wege M. et al. |  |  |
|  | Lundqvist P. et al. |  |  |
|  | Mirlashari J. et al. |  |  |
|  | Nieves HL. |  |  |
|  | Guillaume S. et al. |  |  |
|  | Olshtain-Mann O. et al. |  |  |
|  | Altimier L. |  |  |
|  | Forsythe PL et al. |  |  |
|  | Hagen IH, Iversen VC, Svindseth MF. |  |  |
|  | Vittner D. et al. |  |  |
|  | Chan SH et al. |  |  |
|  | Valizadeh L. et al. |  |  |
|  | Falck AJ et al. |  |  |
|  | Herbst A. et al. |  |  |
|  | Broom M. et al. |  |  |
|  | Reid S. et al. |  |  |
|  | Gay G. et al. |  |  |
|  | Ciupitu-Plath C. et al. |  |  |
|  | Siani SA et al. |  |  |
|  | Lumumba PN. |  |  |
|  | Hemle Jerntorp S. et al. |  |  |
|  | Bry A, Wigert H. |  |  |
|  | Provenzi L. et al. |  |  |
|  | Mazur KM et al. |  |  |
|  | Willem-jan WW et al. |  |  |
|  | Zych B. et al. |  |  |
|  | Orfali K, Gordon E. |  |  |
|  | Aloysius A. et al. |  |  |
|  | Hickson GB et al. |  |  |
|  | Griffith T. et al. |  |  |
|  | Kim AR. |  |  |
|  | Landsem I. et al. |  |  |
|  | Bhandari N. et al. |  |  |
|  | Nassef SK, Blennow M, Jirwe M. |  |  |
|  | Turner MS. |  |  |
|  | Mousavi SS et al. |  |  |
|  | Cheng C. et al. |  |  |
|  | Heidari H, Mardani-Hamooleh M. |  |  |
|  | Zaidi WH et al. |  |  |
|  | Ding X, Zhu L, Zhang R, et al. |  |  |
|  | Alsadaan N. et al. |  |  |
|  | Rosenthal JL et al. |  |  |
|  | Maureen MM et al. |  |  |
|  | Ahern-Lehmann CK. |  |  |
|  | Asai H. |  |  |
|  | Rajabzadeh Z. et al. |  |  |
|  | Barnes S. et al. |  |  |
|  | Fortney CA et al. |  |  |
|  | Dittman K, Hughes S. |  |  |
|  | Fairless HE, Ulloa M. et al. |  |  |
|  | Yu X, Zhang J, Yuan L. |  |  |
|  | Hendricks-Muñoz KD et al. |  |  |
|  | Malusky S. |  |  |
|  | Vetcho S. et al. |  |  |
|  | Logan RM, Dormire S. |  |  |
|  | Schneider J. et al. |  |  |
|  | Janvier A. et al. |  |  |
|  | Johnson AN. |  |  |
|  | Maleki M. et al. |  |  |
|  | Adams SY et al. |  |  |
|  | Beck CT, Vo T. |  |  |
|  | Pouraboli B. et al. |  |  |
|  | Abdeyazdan Z. et al. |  |  |
|  | Dellenmark‐Blom M, Wigert H. |  |  |
|  | Lorié ES et al. |  |  |
|  | Kubicka Z. et al. |  |  |
|  | Lyndon A. et al. |  |  |
|  | Hughes MA et al. |  |  |
|  | Hildingsson IM. |  |  |
|  | Givrad S. et al. |  |  |
|  | Cimke S. |  |  |
|  | Cyr-Alves H, Macken L, Hyrkas K. |  |  |
|  | Meck NE et al. |  |  |
|  | Garne K. et al. |  |  |
|  | Brett J. et al. |  |  |
|  | Arnold L. et al. |  |  |
|  | Michelson KN. |  |  |
|  | Baldoni F, Ancora G, Latour JM. |  |  |
|  | Ichijima E, Kirk R, Hornblow A. |  |  |
|  | Soni R, Tscherning C. |  |  |
|  | Bruton C, Meckley J, Nelson L. |  |  |
|  | Akkaş N, Geçkil E. |  |  |
|  | Aija A. et al. |  |  |
|  | Donohue PK et al. |  |  |
|  | Sigurdson K. et al. |  |  |
|  | Ndango IN. |  |  |
|  | Welch CD, Check J, O’Shea TM. |  |  |
|  | Boucher CA et al. |  |  |
|  | Hendriks MJ, Abraham A. |  |  |
|  | Purdy IB et al. |  |  |
|  | Berns SD et al. |  |  |
|  | Carter JD et al. |  |  |
|  | Aita M, Snider L. |  |  |
|  | Çakmak E, Karaçam Z. |  |  |
|  | Hagen IH, Svindseth MF, Vasset FP. |  |  |
|  | Shahheidari M, Homer C. |  |  |
|  | Wigert H, Berg M, Hellström AL. |  |  |
|  | Franck LS, O'Brien K. |  |  |
|  | Ward FR. |  |  |
|  | Shimizu A, Mori A. |  |  |
|  | Rochefort CM et al. |  |  |
|  | Pados BF, McGlothen-Bell K. |  |  |
|  | Larsson C. et al. |  |  |
|  | Bin-Nun A. et al. |  |  |
|  | Himuro N. et al. |  |  |
|  | Lutz KF. |  |  |
|  | Kasat K, Stoffels G, Ellington M. |  |  |
|  | Gilstrap CM. |  |  |
|  | Joseph RA et al. |  |  |
|  | Musengimana D. |  |  |
|  | Macdonell K. et al. |  |  |
|  | Garten L. et al. |  |  |
|  | Stelwagen MA et al. |  |  |
|  | Yi YZ et al. |  |  |
|  | Rozensztrauch A. et al. |  |  |
|  | Yance B, Do K, Heath J, Fucile S. |  |  |
|  | Wraight CL et al. |  |  |
|  | Whitehill L. et al. |  |  |
|  | LeDuff III LD et al. |  |  |
|  | San Rafael-Gutiérrez S et al. |  |  |
|  | Lakatos PP et al. |  |  |
|  | Fonseca SA et al. |  |  |
|  | Gibson R, Kilcullen M. |  |  |
|  | Brooten D. et al. |  |  |
|  | Asadi Z. et al. |  |  |
|  | Gray JE et al. |  |  |
|  | Newey CR et al. |  |  |
|  | Cartwright K. et al. |  |  |
|  | Patel D. et al. |  |  |
|  | Zhuang Y. et al. |  |  |
|  | Kopsas RE. |  |  |
|  | Dunn MS et al. |  |  |
|  | Niela-Vilén H. et al. |  |  |
|  | Gustafson K. et al. |  |  |
|  | Beal J, Heaman M. |  |  |
|  | Raines DA. |  |  |
|  | Murphy-Oikonen J. et al. |  |  |
|  | Latour JM. |  |  |
|  | McNair C. et al. |  |  |
|  | Taşgıt A, Dil S. |  |  |
|  | Guttmann KF, Orfali K, Kelley AS. |  |  |
|  | Griffin T, Abraham M. |  |  |
|  | Hall SL, Ryan DJ, Beatty J, Grubbs L. |  |  |
|  | Chen H, Dong L. |  |  |
|  | Griffin T. |  |  |
|  | McGowan EC et al. |  |  |
|  | Wang J, He G. |  |  |
|  | Yu X, Zhang J. |  |  |
|  | Yu H, Woo D. et al. |  |  |
|  | Halder P, Bera D, Banerjee A. |  |  |
|  | Miquel-Verges F. et al. |  |  |
|  | Hesham MS et al. |  |  |
|  | Bernaix LW et al. |  |  |
|  | Thomson G. et al. |  |  |
|  | Fugate K. et al. |  |  |
|  | Richards CL. et al. |  |  |
|  | Ahlqvist-Björkroth S et al. |  |  |
|  | Ottosen MJ. |  |  |
|  | Seyed Fatemi N, Haghani H. |  |  |
|  | De Wit S et al. |  |  |
|  | Bloomer MJ. et al |  |  |
|  | Winters R. et al. |  |  |
|  | Resch B. et al. |  |  |
|  | Turner M. et al. |  |  |
|  | Stümpel J. et al. |  |  |
|  | Auslander GK, Netzer D, Arad I. |  |  |
|  | Sarik DA et al. |  |  |
|  | Laudert S. et al. |  |  |
|  | Mariyam M. et al. |  |  |
|  | Alderson P, Hawthorne J, Killen M. |  |  |
|  | Antinora C. et al. |  |  |
|  | Quinn M, Menon U. |  |  |
|  | Gabra MI et al. | Excluded from analysis | They did not meet the inclusion criteria based on their titles and abstracts |
|  | Tuglo LS et al. |  |  |
|  | Thommesen TG. |  |  |
|  | Feleke T. |  |  |
|  | Shibiru S. et al. |  |  |
|  | BHAMBRHO BA et al. |  |  |
|  | Sereshti M. et al. |  |  |
|  | Negasi KB et al. |  |  |
|  | Phiri PG, Chan CW, Wong CL. |  |  |
|  | Nigussie T, Azanaw G, Shumye M. |  |  |
|  | Befekadu L. |  |  |
|  | Mulu GB et al. |  |  |
|  | HASHI MH, Farah AM, Ibrahim SJ. |  |  |
|  | Bazambanza D. |  |  |
|  | Betemariem D. et al. |  |  |
|  | Muktar SA et al. |  |  |
|  | Adane H. |  |  |
|  | Forbes F. et al. |  |  |
|  | Jisso M. et al. |  |  |
|  | Masenya SL. |  |  |
|  | Bedane B. et al. |  |  |
|  | Bayou NB et al. |  |  |
|  | Wassihun T. |  |  |
|  | Manongi R. et al. |  |  |
|  | Hana W. |  |  |
|  | Seniwati T, Wanda D, Nurhaeni N. |  |  |
|  | Bekele B. |  |  |
|  | Aarsland M. |  |  |
|  | Tezera N. et al. |  |  |
|  | Schultz JM, Liptak GS, Fioravanti J. |  |  |
|  | Talbot S. |  |  |
|  | Holdren S, Fair C, Lehtonen L. |  |  |
|  | Griglak S. |  |  |
|  | Chen Y, Zhang J, Bai J. |  |  |
|  | KIRKLAND KA. |  |  |
|  | Cardin AD et al. |  |  |
|  | Richards CL. |  |  |
|  | Abukari AS et al. |  |  |
|  | Ferraresi MF, Arrais AD. |  |  |
|  | Nelson AM, Bedford PJ. |  |  |
|  | JohanneTollofsrud I. et al. |  |  |
|  | Chuo J, Lioy J, Gerdes J. |  |  |
|  | Domanico R. et al. |  |  |
|  | Alves E. et al. |  |  |
|  | Bry AK, Bry A. |  |  |
|  | Dunham MM, Marin T. |  |  |
|  | Helth TD, Jarden M. |  |  |
|  | Im M, Oh J. |  |  |
|  | Mills MM, Sims DC, Jacob J. |  |  |
|  | Baley J. et al. |  |  |
|  | Kropp M. |  |  |
|  | Aftyka A. et al. |  |  |
|  | Pölkki T, Korhonen A, Laukkala H. |  |  |
|  | Trujillo JA et al. |  |  |
|  | Keim MC et al. |  |  |
|  | Tsironi S, Koulierakis G. |  |  |
|  | Mosher S. et al. |  |  |
|  | Yu M, Park CG, Lee S. |  |  |
|  | Edney SK, McHugh G. |  |  |
|  | Brantley AN. |  |  |
|  | Charchuk M, Simpson C. |  |  |
|  | Herini ES, Hartini S, Kusuma MT. |  |  |
|  | Ardal F, Sulman J, Fuller-Thomson E. |  |  |
|  | Hassankhani H. et al. |  |  |
|  | Fanelli S, Zangrandi A. |  |  |
|  | Lakshmanan A. et al. |  |  |
|  | Doede M, Trinkoff AM, Gurses AP. |  |  |
|  | Hua W. et al. |  |  |
|  | Benzies KM. |  |  |
|  | White BR et al. |  |  |
|  | Weber A. et al. |  |  |
|  | Guttmann K, Flibotte J, DeMauro SB. |  |  |
|  | Lima E. |  |  |
|  | Spezia N. et al. |  |  |
|  | Vitner G. |  |  |
|  | Rochefort CM, Clarke SP. |  |  |
|  | Angelhoff C. et al. |  |  |
|  | Seyedfarajollah S. et al. |  |  |
|  | Dol J. et al. |  |  |
|  | Weems MF et al. |  |  |
|  | Shaw RJ et al. |  |  |
|  | Shuman CJ, Morgan M, Vance A. |  |  |
|  | Craig JW. |  |  |
|  | Wielenga JM, Smit BJ, Unk LK. |  |  |
|  | Weyand SA. |  |  |
|  | Alsalem N. et al. |  |  |
|  | Mercado K, Vittner D, McGrath J. |  |  |
|  | Clark OE et al. |  |  |
|  | Parry T. et al. |  |  |
|  | Coughlin K. et al. |  |  |
|  | Smith JR, Pineda RG. |  |  |
|  | McCarley RM et al. |  |  |
|  | Manalu LO, Sutisna MI. |  |  |
|  | Coffman S, Vitt MJ, Deets C. |  |  |
|  | Damrongrak P. et al. |  |  |
|  | Bayisenge M. |  |  |
|  | Yismaw AE, Gelagay AA, Sisay MM. |  |  |
|  | Menalu MM et al. |  |  |
|  | Jebessa S. et al. |  |  |
|  | Araya ES. |  |  |
|  | Amuam DK. |  |  |
|  | Dagnaw FT et al. |  |  |
|  | Berihu G. et al. |  |  |
|  | Jabraeili M. et al. |  |  |
|  | Mengistu BA et al. |  |  |
|  | Delele TG et al. |  |  |
|  | Ahmed Mohammed Sabry F et al. |  |  |
|  | dos Santos Oliveira SJ et al. |  |  |
|  | Ibrahim AM et al. |  |  |
|  | Teklegiorgis H, Ketema H. |  |  |
|  | Tilaye M. |  |  |
|  | Horwood C. et al. |  |  |
|  | Mariano K. et al. |  |  |
|  | Elshanti A. et al. |  |  |
|  | Abie B, Shehibo A, Terefe B. |  |  |
|  | Haile MT. |  |  |
|  | Mbwele B. et al. |  |  |
|  | Badi MB et al. |  |  |
|  | Siva N. et al. |  |  |
|  | Feleke H. et al. |  |  |
|  | Ahmed M. |  |  |
|  | Lulseged S, Deste C. |  |  |
|  | Bayih WA et al. |  |  |
|  | Ayalew K. |  |  |
|  | Heidarzadeh M. et al. |  |  |
|  | Hewedy AA et al. |  |  |
|  | Talus E. et al. |  |  |
|  | Ismail MS, Mohmmed RG. |  |  |
|  | Enjamo M. et al. |  |  |
|  | Kebede AA et al. |  |  |
|  | Permanasari I. et al. |  |  |
|  | Guye AH et al. |  |  |
|  | Ndwiga C. et al. |  |  |
|  | Abdalsemia Elewa A et al. |  |  |
|  | Duresa WB et al. |  |  |
|  | Gonfa DN et al. |  |  |
|  | Endehabtu BF et al. |  |  |
|  | Berhe ET et al. |  |  |
|  | Lulseged S, D'Este C. |  |  |
|  | Billah SM et al. |  |  |
|  | Heen EK et al. |  |  |
|  | Wycliffe S. |  |  |
|  | Mekuria Y, St Denis C. |  |  |
|  | Muwanga E. |  |  |
|  | Wilunda C. et al. |  |  |
|  | Klemming S. et al. |  |  |
|  | Wondie WT et al. |  |  |
|  | Madara CW. |  |  |
|  | NDAGIJIMANA PC. |  |  |
|  | Birtukan A. |  |  |
|  | Ketema DB |  |  |
|  | Akbar S. |  |  |
|  | Gad A. et al. |  |  |
|  | Singh M. et al. |  |  |
|  | Jonsson M. et al. |  |  |
|  | Bonner O. et al. |  |  |
|  | Gehl MB et al. |  |  |
|  | Cricco-Lizza R. |  |  |
|  | McPherson ML et al. |  |  |
|  | Lee YS et al. |  |  |
|  | Burgess E. et al. |  |  |
|  | Altimier L, Phillips R. |  |  |
|  | Acharya S. et al. |  |  |
|  | O’Callaghan N, Dee A, Philip RK. |  |  |
|  | Moratti S. |  |  |
|  | Steven K, Quinn M. |  |  |
|  | Bredemeyer S. et al. |  |  |
|  | Walker HR et al. |  |  |
|  | Goldschmidt KA, Gordin P. |  |  |
|  | van Kampen F. et al. |  |  |
|  | Arabi R, Neill J, Hutton A. |  |  |
|  | Patricia Healy RG, NICU TN. |  |  |
|  | Joseph R, Wellings A, Votta G. |  |  |
|  | Whaley PA, Gosling CG, Schreiner RL. |  |  |
|  | Kahn DJ et al. |  |  |
|  | Lee H, Park JH, Cho H. |  |  |
|  | Day SE. |  |  |
|  | Alinejad-Naeini M. et al. |  |  |
|  | Mainous RO. |  |  |
|  | Stadd K. et al. |  |  |
|  | Bloomer MJ. et al |  |  |
|  | Winters R. et al. |  |  |
|  | Resch B. et al. |  |  |
|  | Turner M. et al. |  |  |
|  | Stümpel J. et al. |  |  |
|  | Auslander GK, Netzer D, Arad I. |  |  |
|  | Sarik DA et al. |  |  |
|  | Laudert S. et al. |  |  |
|  | Mariyam M. et al. |  |  |
|  | Alderson P, Hawthorne J, Killen M. |  |  |
|  | Antinora C. et al. |  |  |
|  | Quinn M, Menon U. |  |  |
|  | Raines DA. |  |  |
|  | Murphy-Oikonen J. et al. |  |  |
|  | Latour JM. |  |  |
|  | McNair C. et al. |  |  |
|  | Taşgıt A, Dil S. |  |  |
|  | Guttmann KF, Orfali K, Kelley AS. |  |  |
|  | Griffin T, Abraham M. |  |  |
|  | Hall SL, Ryan DJ, Beatty J, Grubbs L. |  |  |
|  | Chen H, Dong L. |  |  |
|  | Griffin T. |  |  |
|  | McGowan EC et al. |  |  |
|  | Wang J, He G. |  |  |
|  | Yu X, Zhang J. |  |  |
|  | Yu H, Woo D. et al. |  |  |
|  | Halder P, Bera D, Banerjee A. |  |  |
|  | Miquel-Verges F. et al. |  |  |
|  | Hesham MS et al. |  |  |
|  | Bernaix LW et al. |  |  |
|  | Thomson G. et al. |  |  |
|  | Fugate K. et al. |  |  |
|  | Richards CL. et al. |  |  |
|  | Asadi Z. et al. |  |  |
|  | Gray JE et al. |  |  |
|  | Newey CR et al. |  |  |
|  | Cartwright K. et al. |  |  |
|  | Patel D. et al. |  |  |
|  | Zhuang Y. et al. |  |  |
|  | Kopsas RE. |  |  |
|  | Dunn MS et al. |  |  |
|  | Niela-Vilén H. et al. |  |  |
|  | Gustafson K. et al. |  |  |
|  | Nelson MM. |  |  |
|  | Aliabadi F. et al. |  |  |
|  | Masten M. et al. |  |  |
|  | Turner M, Winefield H. et al. |  |  |
|  | Rinehimer MA. |  |  |
|  | Abbene NM. |  |  |
|  | La Monica LH et al. |  |  |
|  | Abdirahmaan Jacob T. |  |  |
|  | Moreyra A. et al. |  |  |
|  | Donohue PK et al. |  |  |
|  | Sloan K, Rowe J, Jones L. |  |  |
|  | Miller JJ, Serwint JR, Boss RD. |  |  |
|  | Sarin E, Maria A. |  |  |
|  | Akgül Gündoğdu N. et al. |  |  |
|  | Barr P. |  |  |
|  | Gomez DB, Vidal SA, Lima L. |  |  |
|  | Brooten D. et al. |  |  |
|  | Zych B. et al. |  |  |
|  | Orfali K, Gordon E. |  |  |
|  | Aloysius A. et al. |  |  |
|  | Hickson GB et al. |  |  |
|  | Griffith T. et al. |  |  |
|  | Kim AR. |  |  |
|  | Landsem I. et al. |  |  |
|  | Bhandari N. et al. |  |  |
|  | Nassef SK, Blennow M, Jirwe M. |  |  |
|  | Turner MS. |  |  |
|  | Mousavi SS et al. |  |  |
|  | Cheng C. et al. |  |  |
|  | Heidari H, Mardani-Hamooleh M. |  |  |
|  | Zaidi WH et al. |  |  |
|  | Ding X, Zhu L, Zhang R, et al. |  |  |
|  | Alsadaan N. et al. |  |  |
|  | Rosenthal JL et al. |  |  |
|  | Maureen MM et al. |  |  |
|  | Ahern-Lehmann CK. |  |  |
|  | Asai H. |  |  |
|  | Rajabzadeh Z. et al. |  |  |
|  | Barnes S. et al. |  |  |
|  | Fortney CA et al. |  |  |
|  | Dittman K, Hughes S. |  |  |
|  | Fairless HE, Ulloa M. et al. |  |  |
|  | Yu X, Zhang J, Yuan L. |  |  |
|  | Hendricks-Muñoz KD et al. |  |  |
|  | Malusky S. |  |  |
|  | Vetcho S. et al. |  |  |
|  | Logan RM, Dormire S. |  |  |
|  | Schneider J. et al. |  |  |
|  | Janvier A. et al. |  |  |
|  | Johnson AN. |  |  |
|  | Maleki M. et al. |  |  |
|  | Adams SY et al. |  |  |
|  | Beck CT, Vo T. |  |  |
|  | Pouraboli B. et al. |  |  |
|  | Abdeyazdan Z. et al. |  |  |
|  | Dellenmark‐Blom M, Wigert H. |  |  |
|  | Lorié ES et al. |  |  |
|  | Kubicka Z. et al. |  |  |
|  | Lyndon A. et al. |  |  |
|  | Hughes MA et al. |  |  |
|  | Hildingsson IM. |  |  |
|  | Givrad S. et al. |  |  |
|  | Cimke S. |  |  |
|  | Cyr-Alves H, Macken L, Hyrkas K. |  |  |
|  | Meck NE et al. |  |  |
|  | Garne K. et al. |  |  |
|  | Brett J. et al. |  |  |
|  | Arnold L. et al. |  |  |
|  | Michelson KN. |  |  |
|  | Baldoni F, Ancora G, Latour JM. |  |  |
|  | Ding X. et al. |  |  |
|  | Pereira N. et al. |  |  |
|  | Klein V. et al. |  |  |
|  | Ferreira A. et al. |  |  |
|  | Rennick JE et al. |  |  |
|  | Baldoni F. et al. |  |  |
|  | Terp K. et al. |  |  |
|  | Grandjean C. et al. |  |  |
|  | Cruz AC et al. |  |  |
|  | Marini MM et al. |  |  |
|  | Chen, MT. et al. |  |  |
|  | Matalon, C. et al. |  |  |
|  | Nikolaou, D. |  |  |
|  | James, D. E. et al. |  |  |
|  | Savanheimo N. et al. |  |  |
|  | Yoo, J. et al. |  |  |
|  | Çamur Z. et al. |  |  |
|  | Shah PV et al. |  |  |
|  | Spahr CD et al. |  |  |
|  | Zanobini M. et al. |  |  |
|  | Goetting A. et al. |  |  |
|  | Johnston C. et al. |  |  |
|  | Charbonneau E. et al. |  |  |
|  | Friedman BA et al. |  |  |
|  | Sloper P. et al. |  |  |
|  | Shao M. et al. |  |  |
|  | Tilbury C. et al. |  |  |
|  | Guidubaldi J. et al. |  |  |
|  | Byczkowski TL et al. |  |  |
|  | Fantuzzo J. et al. |  |  |
|  | Warde CM et al. |  |  |
|  | Labrie NH et al. |  |  |
|  | Awindaogo F. et al. |  |  |
|  | Misgen M. et al. |  |  |
|  | Govindaswamy P. et al. |  |  |
|  | Dhingra P. et al. |  |  |
|  | Magliyah AF et al. |  |  |
|  | Gavey J. et al. |  |  |
|  | Petteys AR et al. |  |  |
|  | Van Riper M. |  |  |
|  | Mohtashami M. et al. |  |  |
|  | Hames JL et al. |  |  |
|  | Hoge MK et al. |  |  |
|  | Albayrak S. et al. |  |  |
|  | Manning AN. |  |  |
|  | Joseph AM. |  |  |
|  | Gadepalli SK et al. |  |  |
|  | Cheldelin LV et al. |  |  |
|  | Feeley N. et al. |  |  |
|  | Ottosson C. et al. |  |  |
|  | Bruns DA et al. |  |  |
|  | Liu LX et al. |  |  |
|  | Kusnatalia K. et al. |  |  |
|  | Riskin A. et al. |  |  |
|  | Minckas N. et al. |  |  |
|  | Schuler R. et al. |  |  |
|  | Neu M, Klawetter S. et al. |  |  |
|  | Globus O. et al. |  |  |
|  | Ionio C. et al. |  |  |
|  | Thébaud V. et al. |  |  |
|  | Wang L. et al. |  |  |
|  | Toivonen M. et al. |  |  |
|  | Eskandari S. et al. |  |  |
|  | Vazquez V. et al. |  |  |
|  | Jiang S. et al. |  |  |
|  | Matricardi S. et al. |  |  |
|  | Koontz VS. |  |  |
|  | Bertoncelli N. et al. |  |  |
|  | Hall SL et al. |  |  |
|  | Watson J. et al. |  |  |
|  | Carty CL et al. |  |  |
|  | Ribeiro C. et al. |  |  |
|  | Mazlan R. et al. |  |  |
|  | Weis J. et al. |  |  |
|  | Schoenherr J. et al. |  |  |
|  | Judy Levick MS et al. |  |  |
|  | Umberger E. et al. |  |  |
|  | Batton B. et al. |  |  |
|  | Mäkelä H. et al. |  |  |
|  | Swanson JR et al. |  |  |
|  | Enlow E. et al. |  |  |
|  | Franck LS, Oulton K. et al. |  |  |
|  | Beal JA et al. |  |  |
|  | Treherne SC et al. |  |  |
|  | Cooper LG et al. |  |  |
|  | Kenner C. |  |  |
|  | Tiryaki Ö. et al. |  |  |
|  | Kaur M. et al. |  |  |
|  | Schappin R. et al. |  |  |
|  | Lean RE et al. |  |  |
|  | Makkar A. et al. |  |  |
|  | Davidson JE et al. |  |  |
|  | Wege M. et al. |  |  |
|  | Lundqvist P. et al. |  |  |
|  | Mirlashari J. et al. |  |  |
|  | Nieves HL. |  |  |
|  | Guillaume S. et al. |  |  |
|  | Olshtain-Mann O. et al. |  |  |
|  | Altimier L. |  |  |
|  | Forsythe PL et al. |  |  |
|  | Vittner D. et al. |  |  |
|  | Gulo B. et al. | Excluded from analysis | - lacking the reporting of the desired outcomes - studies that had population difference - studies conducted out of the study area |
|  | Mohammed A, Legesse H, Hailu M. |  |  |
|  | Mukeshimana E. et al. |  |  |
|  | Lumumba PN et al. |  |  |
|  | Butt ML et al. |  |  |
|  | Dall'Oglio I. et al. |  |  |
|  | Bastani F. et al. |  |  |
|  | Yılmaz F et al. |  |  |
|  | Tsironi S et al. |  |  |
|  | Lanlehin R. et al. |  |  |
|  | Voulgaridou A. et al. |  |  |
|  | Srivastava R. et al. |  |  |
|  | Jayasinghe C. et al. |  |  |
|  | McCormick MC et al. |  |  |
|  | Lake ET et al. |  |  |
|  | Conner JM et al. |  |  |
|  | Sankar V. et al. |  |  |
|  | Hagen IH et al. |  |  |
|  | Campus MN et al. |  |  |
|  | Nguyen AT et al. |  |  |
|  | Salehi Z. et al. |  |  |
|  | Mitchell-DiCenso A. et al. |  |  |
|  | Latour JM et al. |  |  |
|  | Lilo EA et al. |  |  |
|  | Demis A. et al. |  |  |
|  | Silesh M. et al. |  |  |
|  | Yifru Berhan YB et al. |  |  |
|  | Kidane A. et al. |  |  |
|  | Oude Maatman SM et al. |  |  |
|  | Latour JM, Hazelzet JA et al. |  |  |
|  | Tiryaki O. et al. |  |  |
|  | Ullsten A. et al. |  |  |
|  | Ferentzi H. et al. |  |  |
|  | Cleveland LM. et al. |  |  |
|  | De Bernardo G. et al. |  |  |
|  | Russell G. et al. |  |  |
|  | Abuqamar M. et al. |  |  |
|  | Ebrahim S. et al. |  |  |
|  | Stevens DC et al. |  |  |
|  | Adama EA et al. |  |  |
|  | Mol C. et al. |  |  |
|  | Heidari H. et al. |  |  |
|  | Jannes C. et al. |  |  |
|  | Voos KC et al. |  |  |
|  | Shrestha T, Bista AP, Shrestha S. |  |  |
|  | Obeidat HM et al. |  |  |
|  | Kadivar M. et al. |  |  |
|  | Cintra CD et al. |  |  |
|  | Liu TT et al. |  |  |
|  | Zorro C. et al. |  |  |
|  | Ding X. et al. |  |  |
|  | Pick V. et al. |  |  |
|  | Dhingra P. |  |  |
|  | Fazio SB et al. |  |  |
|  | Shahheidari M. et al. |  |  |
|  | Reis MD et al. |  |  |
|  | Orfali K. et al. |  |  |
|  | Abuidhail J. et al. |  |  |
|  | Koontz VS et al. |  |  |
|  | Williams KG et al. |  |  |
|  | Weiss S. et al. |  |  |
|  | Moore KA et al. |  |  |
|  | Ladani MT et al. |  |  |
|  | Gallagher K. et al. |  |  |
|  | Kasat K. et al. |  |  |
|  | Haines C. et al. |  |  |
|  | Shafer JS et al. |  |  |
|  | Rey JM et al. |  |  |
|  | Summers JA et al. |  |  |
|  | Ygge BM et al. |  |  |
|  | Williams G. et al. |  |  |
|  | Hart CN et al. |  |  |
|  | Pelchat D. et al. |  |  |
|  | Schaffer P. et al. |  |  |
|  | Acs G. et al. |  |  |
|  | Kruszecka-Krówka A. et al. |  |  |
|  | Gibbons S. et al. |  |  |
|  | Mbuvi John B. |  |  |
|  | Galanis P. et al. |  |  |
|  | Segers E. et al. |  |  |
|  | Nieves H. et al. |  |  |
|  | Sakonidou S. et al. |  |  |
|  | Martin AE et al. |  |  |
|  | Sánchez-Veracruz MJ et al. |  |  |
|  | Finlayson K. et al. |  |  |
|  | Cescutti‐Butler L. et al. |  |  |
|  | Brødsgaard A. et al. |  |  |
|  | Rihan SH et al. |  |  |
|  | Saxton SN et al. |  |  |
|  | Epstein EG et al. |  |  |
|  | Ghadery-Sefat A. et al. |  |  |
|  | Meert KL et al. |  |  |
|  | Ranchod TM et al. |  |  |
|  | Sikorova L. et al. |  |  |
|  | Franck LS et al. |  |  |
|  | Kjellsdotter A. et al. |  |  |
|  | Zhang R et al. |  |  |
|  | Fotiou C. et al. |  |  |
|  | Smith VC et al. |  |  |
|  | Ramezani T. et al. |  |  |
|  | Holditch-Davis D. et al. |  |  |
|  | Abdel-Latif ME et al. |  |  |
|  | Heinemann AB et al. |  |  |
|  | Nazari R. et al. |  |  |
|  | Oliveira AR et al. |  |  |
|  | Skene C. et al. |  |  |
|  | Zauche LH et al. |  |  |
|  | Wigert H. et al. |  |  |
|  | Baylis R. et al. |  |  |
|  | Lantz B. et al. |  |  |
|  | Serlachius A. et al. |  |  |
|  | Rosenthal SA et al. |  |  |
|  | Tran C. et al. |  |  |
|  | Koliouli F, Gaudron CZ. |  |  |
|  | Balbino FS et al. |  |  |
|  | Raiskila S. et al. |  |  |
|  | Gooding JS et al. |  |  |
|  | Sweet L. et al. |  |  |
|  | Murthy P. et al. |  |  |
|  | Penticuff JH et al. |  |  |
|  | Tandberg BS et al. |  |  |
|  | Turan T. et al. |  |  |
|  | Williams L. et al. |  |  |
|  | Giuseppe DB et al. |  |  |
|  | Mills MM et al. |  |  |
|  | Hagen IH, Iversen VC, Svindseth MF. |  |  |
|  | Chan SH et al. |  |  |
|  | Valizadeh L. et al. |  |  |
|  | Falck AJ et al. |  |  |
|  | Herbst A. et al. |  |  |
|  | Broom M. et al. |  |  |
|  | Reid S. et al. |  |  |
|  | Gay G. et al. |  |  |
|  | Ciupitu-Plath C. et al. |  |  |
|  | Siani SA et al. |  |  |
|  | Lumumba PN. |  |  |
|  | Hemle Jerntorp S. et al. |  |  |
|  | Bry A, Wigert H. |  |  |
|  | Provenzi L. et al. |  |  |
|  | Mazur KM et al. |  |  |
|  | Willem-jan WW et al. |  |  |
|  | Czarnecki ML et al. |  |  |
|  | van Veenendaal NR et al. |  |  |
|  | Hoeben H. et al. |  |  |
|  | Jayasinghe C, Abeysena C. |  |  |
|  | Lebel V. et al. |  |  |
|  | Negarandeh R. et al. |  |  |
|  | Rosenthal SA, Nolan MT. |  |  |
|  | Akmali P. et al. |  |  |
|  | Caeymaex L. et al. |  |  |
|  | Kainiemi E. et al. |  |  |
|  | Campbell-Yeo M et al. |  |  |
|  | Garne Holm K. et al. |  |  |
|  | Young C. et al. |  |  |
|  | Grunberg VA et al. |  |  |
|  | Levick J. et al. |  |  |
|  | De Rouck S, Leys M. |  |  |
|  | Maria A. et al. |  |  |
|  | Wang LL et al. |  |  |
|  | Berman L. et al. |  |  |
|  | Bourque CJ et al. |  |  |
|  | Taneja R, Batra P, Sadiza J. |  |  |
|  | Loewenstein K, Barroso J, Phillips S. |  |  |
|  | Stutts A. |  |  |
|  | Lester BM et al. |  |  |
|  | Macho P. |  |  |
|  | Cortezzo DE et al. |  |  |
|  | Hofbauer JM et al. |  |  |
|  | Ganguly R. et al. |  |  |
|  | Rafiey H. et al. |  |  |
|  | Flacking R, Breili C, Eriksson M. |  |  |
|  | Grosik C. et al. |  |  |
|  | Spruill CT, Heaton A. |  |  |
|  | Modé RI et al. |  |  |
|  | Arshadi Bostanabad M et al. |  |  |
|  | Biasini A. et al. |  |  |
|  | Yager PH et al. |  |  |
|  | Razavi Nejad M. et al. |  |  |
|  | Martinez AM et al. |  |  |
|  | Klawetter S. et al. |  |  |
|  | Fucile S, Samson J, Rockley J, Dow K. |  |  |
|  | Garfield CF, Lee Y, Kim HN. |  |  |
|  | Lantz B, Ottosson C. |  |  |
|  | Rabie D. et al. |  |  |
|  | Rio L, Fadda MD et al. |  |  |
|  | Bua J. et al. |  |  |
|  | Aucott S. et al. |  |  |
|  | Kowalski WJ et al. |  |  |
|  | Arriaga-Redondo M et al. |  |  |
|  | Larocque C. et al. |  |  |
|  | Gonya J. et al. |  |  |
|  | Hamel MN, Beltran SJ. |  |  |
|  | Shirazi ZH et al. |  |  |
|  | O’Shea TM. |  |  |
|  | Waldron MK. |  |  |
|  | Almutairi A, Gavine A, McFadden A. |  |  |
|  | Jones L, Woodhouse D, Rowe J. |  |  |
|  | Drago M. et al. |  |  |
|  | Juretschke LJ et al. |  |  |
|  | Edéll‐Gustafsson U et al. |  |  |
|  | Buek KW, Cortez D, Mandell DJ. |  |  |
|  | Rosenthal SA. |  |  |
|  | Brelsford G. et al. |  |  |
|  | Sims DC et al. |  |  |
|  | Burrows A. |  |  |
|  | Locale AU et al. |  |  |
|  | Kasparian NA et al. |  |  |
|  | De Souza D et al. |  |  |
|  | Mardani-Hamooleh M, Heidari H. |  |  |
|  | Abukari AS, Schmollgruber S. |  |  |
|  | Partridge JC et al. |  |  |
|  | McCord H. et al. |  |  |
|  | Ansari NS et al. |  |  |
|  | Bosch S, Bledsoe T, Jenzarli A. |  |  |
|  | Granrud MD et al. |  |  |
|  | Meesters N. et al. |  |  |
|  | Dosani A. et al. |  |  |
|  | Frize M, Bariciak E, Gilchrist J. |  |  |
|  | Akard TF et al. |  |  |
|  | Schecter R. et al. |  |  |
|  | Weyand SA et al. |  |  |
|  | Altimier L, Kenner C, Damus K. |  |  |
|  | Glazer KB et al. |  |  |
|  | Thibodeau BA et al. |  |  |
|  | Beal J, Heaman M. |  |  |
|  | Ichijima E, Kirk R, Hornblow A. |  |  |
|  | Soni R, Tscherning C. |  |  |
|  | Bruton C, Meckley J, Nelson L. |  |  |
|  | Akkaş N, Geçkil E. |  |  |
|  | Aija A. et al. |  |  |
|  | Donohue PK et al. |  |  |
|  | Sigurdson K. et al. |  |  |
|  | Ndango IN. |  |  |
|  | Welch CD, Check J, O’Shea TM. |  |  |
|  | Boucher CA et al. |  |  |
|  | Hendriks MJ, Abraham A. |  |  |
|  | Purdy IB et al. |  |  |
|  | Berns SD et al. |  |  |
|  | Carter JD et al. |  |  |
|  | Aita M, Snider L. |  |  |
|  | Çakmak E, Karaçam Z. |  |  |
|  | Hagen IH, Svindseth MF, Vasset FP. |  |  |
|  | Shahheidari M, Homer C. |  |  |
|  | Wigert H, Berg M, Hellström AL. |  |  |
|  | Franck LS, O'Brien K. |  |  |
|  | Ward FR. |  |  |
|  | Shimizu A, Mori A. |  |  |
|  | Rochefort CM et al. |  |  |
|  | Pados BF, McGlothen-Bell K. |  |  |
|  | Larsson C. et al. |  |  |
|  | Bin-Nun A. et al. |  |  |
|  | Himuro N. et al. |  |  |
|  | Lutz KF. |  |  |
|  | Kasat K, Stoffels G, Ellington M. |  |  |
|  | Gilstrap CM. |  |  |
|  | Joseph RA et al. |  |  |
|  | Musengimana D. |  |  |
|  | Macdonell K. et al. |  |  |
|  | Garten L. et al. |  |  |
|  | Stelwagen MA et al. |  |  |
|  | Yi YZ et al. |  |  |
|  | Rozensztrauch A. et al. |  |  |
|  | Yance B, Do K, Heath J, Fucile S. |  |  |
|  | Wraight CL et al. |  |  |
|  | Whitehill L. et al. |  |  |
|  | LeDuff III LD et al. |  |  |
|  | San Rafael-Gutiérrez S et al. |  |  |
|  | Lakatos PP et al. |  |  |
|  | Fonseca SA et al. |  |  |
|  | Gibson R, Kilcullen M. |  |  |
|  | Wong SE et al. |  |  |
|  | Auslander GK et al. |  |  |
|  | Hill C. et al. |  |  |
|  | Kolmakova D. |  |  |
|  | Hendrawati S. et al. |  |  |
|  | Risanger LI et al. |  |  |
|  | De Rouck S. et al. |  |  |
|  | Enke C. et al. |  |  |
|  | Mardani-Hamooleh M. et al. |  |  |
|  | Baughcum AE et al. |  |  |
|  | Al-Motlaq MA et al. |  |  |
|  | Lam JY et al. |  |  |
|  | Sng QW et al. |  |  |
|  | Foster J. et al. |  |  |
|  | Spezia N. et al. |  |  |
|  | Profit J. et al. |  |  |
|  | Blackington SM et al. |  |  |
|  | Garfield CF et al. |  |  |
|  | Browne JV et al. |  |  |
|  | Ahlqvist-Björkroth S et al. |  |  |
|  | Ottosen MJ. |  |  |
|  | Seyed Fatemi N, Haghani H. |  |  |
|  | De Wit S et al. |  |  |
|  | Dien R. et al. |  |  |
|  | Hutchinson SW. |  |  |
|  | Soleimani F. et al. |  |  |
|  | Kynø NM et al. |  |  |
|  | Melançon J et al. |  |  |
|  | Byers JF et al. |  |  |
|  | Chung RK, Kim UO, Basir MA. |  |  |
|  | Baghlani R. et al. |  |  |
|  | Labree W. et al. |  |  |
|  | Hamel M. |  |  |
|  | Shanty L. et al. |  |  |
|  | D'Agata AL, McGrath JM. |  |  |
|  | Piris-Borregas S et al. |  |  |
|  | Walker SH et al. |  |  |
|  | Crisp L. |  |  |
|  | Rambod M. et al. |  |  |
|  | Kodjebacheva GD et al. |  |  |
|  | Ali Z. |  |  |
|  | Redd L. et al. |  |  |
|  | Ocampo MJ et al. |  |  |
|  | Tarja P. et al. |  |  |
|  | Murray CH, Joseph RA. |  |  |
|  | Hellmann J. et al. |  |  |
|  | Meredith JL, Jnah A, Newberry D. |  |  |
|  | Mizutani N. |  |  |
|  | Dahan S. et al. |  |  |
|  | Golec L. et al. |  |  |
|  | Joshi P. et al. |  |  |
|  | Widger K, Picot C. |  |  |
|  | Demel A. |  |  |
|  | Barsman SG. |  |  |
|  | Anderson R. et al. |  |  |
|  | Bardach SH et al. |  |  |
|  | Gilmer MJ et al. |  |  |
|  | Stefana A. et al. |  |  |
|  | Lakhani J. et al. |  |  |
|  | Blackington SM, McLauchlan T. |  |  |
|  | Shah V, Warre R, Lee SK. |  |  |
|  | de Castro KN et al. |  |  |
|  | Mimani Minuta W. et al. |  |  |
|  | Workneh R. |  |  |
|  | Mahon P. et al. |  |  |
|  | Reid T. et al. |  |  |
|  | Hurst I. et al. |  |  |
|  | Saunders RP et al. |  |  |
|  | Carter BS et al. |  |  |
|  | Hallowell SG et al. |  |  |
|  | Steedman WK. |  |  |
|  | Mengesha EW et al. |  |  |
|  | Lin M. et al. |  |  |
|  | Clarke-Pounder JP et al. |  |  |
|  | Marçola L. et al. |  |  |
|  | Maram Abuqamar RN et al. |  |  |
|  | Doede M. et al. |  |  |
| **Studies met the inclusion criteria and considered suitable for the final meta-analysis** | | | |
| S.No | Authors | URL for unpublished articles | |
|  | Fikadu L. et al. |  |  |
|  | Jamie AH et al. |  |  |
|  | Berhan Y. |  |  |
|  | Ali MS. et al. |  |  |
|  | Alle YF et al. |  |  |
|  | Alemu A. et al. |  |  |
|  | Endale H. | http://thesisbank.jhia.ac.ke/id/eprint/6410 | |
|  | Sileshi E et al. |  |  |
|  | Adal Z et al. |  |  |
|  | Mekonnen WN et al. |  |  |
|  | Workie M et al. | http://ir.haramaya.edu.et//hru/handle/123456789/7216 | |
