# Supplementary material for: Parental satisfaction towards care given at neonatal intensive care unit in Ethiopia: A systematic review and meta-analysis
Source: PLoS One. 2024 Dec 5;19(12):e0313451. doi: 10.1371/journal.pone.0313451 (PMC11620403; doi:10.1371/journal.pone.0313451)
Supplement: Attachment — Submitted filename: Response to Reviewers.docx [file pone.0313451.s008.docx]

**Date: August 11, 2024**

**Subject:** Response to editor, and reviewers for the revised manuscript submission.

**Title:** Parental satisfaction towards care given at neonatal intensive care unit in Ethiopia: a systematic review and meta-analysis; [PONE-D-24-18838]

**Dear Editor and Reviewers,**

We are grateful for your careful reading of our work and your insightful comments. We truly value the time and energy you invested in offering thoughtful feedback and recommendations. Every one of your comments has been thoroughly reviewed, and the appropriate changes have been implemented in response. The individual reviewer comments have been addressed by explaining the adjustments made and offering a thorough response to each comment. We have also considered the journal requirements and ensured that our paper conforms to all relevant guidelines and formatting requirements. We believe that these changes have greatly improved our study's quality, precision, and clarity. We are certain that the revised manuscript now successfully addresses the issues brought up throughout the review process and complies with the journal's requirements. We would want to thank you again for all of your helpful advice and suggestions during this process. We welcome any further comments or advice you may have.

Thank you for your time and consideration.

Sincerely,

The authors

Find my suggestions here below:

Indicate the PROSPERO Registration Number:

Clearly state the PROSPERO registration number for this systematic review and meta-analysis (SRMA) in the manuscript.

***Response:*** *We appreciate your insightful comment. We apologize for the missing PROSPERO registration number in the previous submission. The updated manuscript now includes the PROSPERO registration number (CRD42024570971) on page number 2 and 5, line number 33 and 110. We thank you for your valuable comment.*

Use Appropriate Regional Nomenclature:

Ensure that the appropriate and correct naming of regions within the respective country is consistently used throughout the manuscript. This includes all text references, figures (such as Figure 4), tables, and any supplementary materials. for example Oromo Vs. Oromia, Harar Vs. Harari, Southern Vs. SNNPR etc.

***Response:*** *We value your meticulous attention to detail. We have carefully reviewed the manuscript to ensure consistency in the naming of regions. We have appropriately changed all text references, figures, tables, and supplemental materials in the amended document. We believe these changes improve the manuscript's quality.*

Detailed Results Section in Abstract is needed:

Expand the results section of the abstract to provide more detailed and engaging information. Include key findings, statistical significance, and any important trends or patterns observed in the data. This will make the abstract more informative and appealing to the audience.

***Response:*** *We appreciate your insightful recommendation to make the results part of the abstract longer. We have meticulously rewritten the abstract to incorporate more detailed findings, including key results, statistical significance, and salient trends (page number 2, lines 39-41). We have tried to present a thorough synopsis of the study, but we have strictly followed the journal's 300-word limit. Thank you once again for your insightful suggestions.*

Change Subtopic Title:

Change the title of the subtopic from "Result" to "Results" to reflect the content accurately.

***Response:*** *We appreciate your recommendation to change the subtopic title from "Result" to "Results." This modification has been made to correctly reflect the section's content in the revised manuscript.*

**Reviewer #1:** Thanks to the authors of the manuscript for trying to synthesize evidence on patient satisfaction in NICU. In my review, I have found some methodological issues and I have organized my feedback chronological to the chapters and subchapters of this manuscript. Please find the followings:

1) Abstracts

1.1: In the abstract's method section, if we have any word limitation then, I would prefer mentioning about the risk of bias of this SRMA, rather than mentioning which software we use to extract data or analyse the data.

***Response:*** *We are grateful for your recommendation to prioritize information in the abstract's methods section. Following the journal's rigorous 300-word limit, we have concentrated on an assessment of the risk of bias in the revised manuscript (lines 31-33). Thank you once again for your valuable suggestions.*

2) Introduction

2.1: It is ambitious to say, this the first kind of study, I'd prefer rephrasing this sentence as this study might be the extension of the available evidence. Also, I just found one published SRMA on the similar topic from Ethiopia (<https://www.ncbi.nlm.nih.gov/pmc/articles/PMC11000344/>).

***Response:*** *We value the insightful reference you provided (*[*https://www.ncbi.nlm.nih.gov/pmc/articles/PMC11000344*](https://www.ncbi.nlm.nih.gov/pmc/articles/PMC11000344)*) and your astute observation. The statement has been updated to reflect the previous studies. Our study expands on the body of knowledge by performing a more thorough systematic review and meta-analysis (SRMA), incorporating, a more comprehensive view of the data available compared to previous studies like the one mentioned, which included a small number of articles with a smaller sample size.*

3) Methods

3.1: Language Restriction: Including only English articles in the era of internet, AI, google translators, proves your literature search might miss some of the important articles published in other language.

***Response:*** *We really acknowledge your concern regarding the potential limitations of restricting the search to publications written in the English language. Although we can't rule out the potential of overlooking pertinent research written in other languages, a careful review of Ethiopia's body of literature on the subject turned up no research written in any language other than English (page number 7, lines 138-139). We used the available translation tools to perform preliminary searches in Ethiopian languages in order to further address your concern. There were no further related studies found by these searches. We believe that our thorough search strategy, including the exploration of non-English literature, sufficiently handles the possibility of any pertinent information being overlooked.*

3.2: Exclusion criteria: Excluding articles that did not report on the prevalence of parental satisfaction is an inappropriate approach for this type of SRMA where the main focus is on patient satisfaction. Most of the studies use different scales to report patient satisfaction and they have different levels of reporting. Even for this particular research, all the included studies did not use the same tool to assess the patient satisfaction. I would suggest the authors to read Cochrane SRMA handbook and their published SRMA to have a clear idea how to report SRMAs with different scales and different reporting system.

***Response:*** *We appreciate your insightful suggestions on the exclusion criteria. After giving your idea great thought, we have updated our exclusion criteria in the revised manuscript (****page number 7, lines 141-144****). We have included all types of observational study designs in our study. To guarantee the validity of our study, we have followed the recommendations made in the Cochrane Handbook for Systematic Reviews of Interventions. Again, we are very grateful for your review.*

3.3: Exclusion Criteria: How did the authors define, methodological problems, such as inadequate sample size, sampling techniques, incomplete data, inadequate statistical analysis, or any other relevant methodological limitations? What are their thresholds of exclusion? This needs to be mentioned in the Method section clearly.

***Response:*** *We thank you for your meticulous attention to methodological rigor. We have updated our exclusion criteria in the revised manuscript (****page number 7, lines 141-144****). In the updated manuscript, we have corrected typographical errors and believe these changes improve the soundness of the exclusion criteria.*

3.4: The Holy 2012 tool can be used to assess the Risk of bias of the prevalence based articles, it doest not assess the internal and external validity of the articles. Please omit or rephrase the sentence.

***Response:*** *We appreciate and value your recommendation to make the Holy 2012 tool's scope clearer. The sentences have been changed in the updated manuscript (page number 8, lines 165-166). Thank you once again for your recommendation.*

3.5: Why the authors assess Risk of Bias using two different scales (NOQAS and Holy tool) since authors only included the prevalence studies for this SRMA?

***Response:*** *We really appreciate your feedback and value your thorough assessment of our manuscript. Although the review primarily concentrates on the prevalence of parental satisfaction, it is crucial to remember that we also explored associated factors that may have an impact on the prevalence. We used the Holy tool especially to measure the risk of bias and the NOQAS tool to evaluate the quality of each study in order to completely analyze the quality of the included studies. The selection of NOQAS was based on its popularity as a tool for evaluating the quality of observational studies. Conversely, the Holy Tool was applied with greater specificity to articles that were prevalence-based. We were able to offer a thorough assessment of the included studies by making use of both approaches. We believe that using both approaches strengthens the quality of our findings.*

3.6: Statistical analysis: Authors are reporting prevalence of satisfaction, it is unclear why authors have done the meta-regression here.

***Response:*** *We appreciate your thoughtful assessment of our statistical analysis and your comments. Our study explores associated factors that affect parental satisfaction in addition to the prevalence of parental satisfaction, which is our main finding. To investigate the effect of these characteristics on the prevalence of parental satisfaction across the included studies, meta-regression was utilized. In order to investigate possible sources of heterogeneity in the pooled prevalence, we also performed meta-regression. Once again, we are very grateful for your review.*

3.7: GRADE assessment is an important tool to assess the certainty of evidence of your meta-analysis, which authors skipped. I'd suggest add this to the manuscript.

***Response:*** *We appreciate your insightful suggestion. We agree that GRADE assessment is important for determining the certainty of evidence in meta-analysis. We have updated the manuscript with a thorough GRADE assessment based on your suggestion. The GRADE assessment is now included in the methods section, statistical analysis subsection (****page number 9, lines 187–190****), where we provide a thorough review of the certainty of evidence for each outcome. We believe that this improvement greatly increases the transparency and rigor of our study. Once again, we are very grateful for your valuable suggestions.*

4) Results

4.1: What did the authors do since they got very high heterogeneity? They did mention they explored their heterogeneity through subgroup analysis. However, in their forest plots, i did not find and I^2 value for the subgroups, which is very perplexing for the authors. They did report I^2 value in their "Results: Subgroup Analysis" section; however, I believe authors have interpreted the heterogeneity in their subgroup analysis incorrectly. The I^2 = 90.45% is the overall heterogeneity. Please follow the Cochrane SRMA handbook or other available literatures.

***Response:*** *We understand that the level of heterogeneity in our meta-analysis concerns you. Because of the diverse geographical and socio-economic situations across different regions of Ethiopia, it is expected that there will be variations in the outcomes. To make sense of this, we performed sensitivity analysis and meta-regression in addition to subgroup analysis to investigate possible sources of heterogeneity. However, the result for meta-regression was insignificant, and sensitivity analysis also indicated that no study had a significant impact on the overall pooled estimated prevalence as this is presented in our manuscript (****page number 13, lines 262-269****). In the amended manuscript, we have added I^2^ values for every subgroup analysis to the forest plots, as advised. In the event that our result had very high heterogeneity, we would write this as a limitation in our manuscript. Once again, we sincerely appreciate your contribution in bringing these concerns to our attention, and we value your efforts to improve the quality of our study.*

4.2: For the forest plots, please try to add the I^2 values for each of the subgroups, chi-square statistics, and p-value.

***Response:*** *We thank you for your attention to detail and your suggestions on how to make our manuscript better. Your request to include chi-square statistics in the forest plots has been carefully reviewed. But as you correctly pointed out, our analysis includes 11 articles across five geographical regions of Ethiopia, with a maximum of four studies conducted in the Amhara region and a minimum of one study in the Southern Nations, Nationalities, and Peoples' Region. As chi-square statistics are usually used to evaluate heterogeneity among multiple studies within a subgroup, this particular feature of our dataset makes them challenging to compute. To address your concerns, we have added I² values for each subgroup and the overall p-value in the forest plots. However, the calculated I² values and p-values were (I^2 values =. % and p-value =.) as shown in the subgroup analysis (Figures 4 and 5). This is due to the limited number of studies (three or fewer) in each region or year. We would be pleased to offer more information or analysis if necessary, and we are still available for additional clarification on this topic. Again, thank you so much for your valuable feedback.*

**Reviewer #2:** Review Reports

Title: Parental satisfaction towards care given at neonatal intensive care unit in Ethiopia: a systematic review and meta-analysis Manuscript Number: PONE-D-24-18838

Comments

• The issues is contemporary agenda.

***Response:*** *Thank you for recognizing the study's relevance to current research objectives. In NICUs across the globe, parent satisfaction with newborn care is a vital component of quality improvement. In particular, the Ethiopian context, where improving parental experiences is desperately needed, is the subject of this study.*

• The background fails to entail all what it intends to entail. Avoid repeated use of sentences. E.g. Adverse consequences of dis-satisfaction.

***Response:*** *We appreciate your feedback. With great effort, the background section was created to offer a thorough synopsis of the pertinent literature. We believe the details provided are adequate to illustrate the background and purpose of the research. We have carefully examined the section and made the required changes to improve its coherence and clarity. We have also avoided the repetition of sentences in the revised manuscript. Again, Thank you for your insightful comment.*

• The methods fail to describe whether the inclusion of unpublished articles and its where about.

***Response:*** *We appreciate your valuable feedback. This systematic review contained unpublished articles. We looked through the grey literature in great detail to find relevant studies. The search approach used to find unpublished articles is also clearly described in the methods section, together with information on the databases and sources that were looked for (page number 5, lines 106 and 113). We have carefully gone over this section to make sure all of the information is accurate and thorough. Thanks once again for your valuable comment.*

• The extractors, ambiguity an dhow the ambiguity was solved as well as the inclusion of retracted publication is not mentioned.

***Response:*** *We really appreciate your comment. The data extraction process is described in detail in the methods section (page number 7, lines 146–151), together with information about the extractors' identities and ambiguity-resolution techniques (lines 125-128). We also clearly indicate that papers that were retracted were not included in the analysis. We have gone over this section to make sure all of the information is given in an understandable and thorough manner.*

• The **PCO** is not described

***Response:*** *We appreciate your feedback. Our revised manuscript now clearly states the PCO framework (page number 6-7, lines 133-136). The study's population, intervention, comparison, and outcome have all been precisely defined. We are grateful for bringing this to our attention and for your suggestions on improving the clarity of our study.*

• The result and the discussion should be brief and clear.

***Response:*** *We appreciate your review and insightful suggestions. The results and discussion sections have undergone a thorough review process to guarantee that the study's findings are communicated in an understandable and efficient manner. We believe the way it is currently presented is the best way to communicate the main findings of the study.*

• The discussion should be relevant contextual and explanatory.

***Response:*** *Thank you so much for your review and insightful feedback. The discussion section has been carefully revised to improve its explanatory power, relevance, and context. We have given a more thorough examination of the implications of our results and improved the link between our findings and the body of current literature. These changes, in our opinion, greatly raise the discussion's general caliber.*

• Edit for language and grammar E.g. Use ‘Oromia’ instead of Oromo.

***Response:*** *We appreciate your review and insightful suggestions. Our manuscript has been thoroughly examined for grammar and language errors, and the required modifications have been made, such as changing "Oromo" with "Oromia" as suggested. We value your meticulous attention to detail.*

**Reviewer #3:** Parental satisfaction towards care given at neonatal intensive care unit in Ethiopia: a systematic review and meta-analysis

Reviewer Comment:

Thank you. I am pleased to review the following manuscript. I hope incorporating the following comment would enhance the quality of the manuscript at all.

Thank you.

Abstract:
1. The method section should contain the timeframe of relevant published articles.

***Response:*** *We value your recommendation to add the timeframe of relevant published articles in the abstract methods section. However, we were unable to include this information in the abstract because of the journal's strict word limit (no more than 300 words). We believe that the study's objectives, methods, key findings, and conclusions are succinctly described in the abstract. We kindly point you to the methods section, where the timeframe of relevant published articles is explained in detail, for a thorough comprehension of the literature search (page number 7, line 140). We believe this approach gives the main text the required depth without sacrificing the abstract's clarity and succinctness.*

2. The result should reflect the aim thus some important relevant associated factors should be present.

***Response:*** *We appreciate your review and insightful suggestions. We agree that the results should clearly reflect the study's aims. We have carefully reviewed our manuscript and included additional associated factors to improve interpretation of our findings in our amended manuscript (page 2 and lines 39-41). We are grateful for bringing this to our attention and for your suggestions on improving the clarity of our study.*

3. There is some inconsistency or may the author could not reflect which tried to express in the conclusion section 42-44 line. Try to simplify the line.

***Response:*** *We thank you for carefully reviewing the manuscript. We agree that the phrasing wording in lines 42–44 of the conclusion section could be improved. To make the language more succinct and clearer, we updated it in the revised manuscript (pages 2–3, lines 43–46). Once again, we appreciate you bringing this to our attention and helping to improve the clarity and presentation of our study.*

4. PROSPERO Reg. number is missing. Without this Systematic review is incomplete.

***Response:*** *We appreciate your insightful comment. We apologize for the missing PROSPERO registration number in the previous submission. The updated manuscript now includes the PROSPERO registration number (CRD42024570971) on page number 2 and 5, lines 33 and 110. We believe that this addition improves our study’s transparency and reproducibility.*

Introduction:
1. Try to rephrase the 47 line its just a copy of abstract.

***Response:*** *We thank you for your meticulous attention to detail. To reduce duplication and offer more explanation, we have altered the sentences in the updated manuscript (page number 3, line 49). Once again, we are very grateful for your review.*

2. According to line 50, WHO reference is not appropriately given.

***Response:*** *We thank you for your careful review of our manuscript. We have carefully considered your concern regarding the WHO reference on line 50 and made the necessary corrections in light of it in our updated manuscript. Again, thank you for your insightful comments.*

3. Although the introduction section is well written but I think it has missing some important factors like, defining neonatal period, describing the existing NICU care given prevalence in other countries. Being a review paper, it should explain how patient satisfaction is important to reduce IMR using data.

***Response:*** *We appreciate your thorough review of our manuscript and value the time and effort you invested in giving us feedback to improve our work. The neonatal period is defined in our amended manuscript based on your suggestion (page number 3, lines 51-52). Rather than focusing on the occurrence of NICU care, this study particularly examines parental satisfaction with NICU care. Consequently, even though we recognize the significance of this data, it is not within the purview of our present study. We have spent a good deal of space in our introduction (lines 59–66 and 84–91 on pages 3, 4, and 5) talking about how important parental satisfaction is to lowering the infant mortality rate. We have provided relevant data to support this claim. Thank you again for your valuable feedback.*

Methods and materials:

1. The search term should contain “father’s satisfaction”, “Ethiopia” should be specified using breakdown of southern part or northern part.

***Response:*** *We appreciate your insightful feedback regarding the search terms we used in our study. With great attention, we have updated the search terms to include "father's satisfaction" and have given the precise geography of Ethiopia by naming each region. The updated manuscript now includes these modifications (page number 6, lines 114-119). We believe these changes improve our search strategy's precision and coverage. Once again, we appreciate your insightful suggestion.*

2. In the study selection process, three researchers review but what about the disagreements was how resolved? It is not described at all. What about the Full text screening process?

***Response:*** *We appreciate your thorough reading of our manuscript. We have considered your comments regarding the study selection process. The process for settling disputes amongst the three reviewers during the title/abstract and full-text screening processes has been explained in detail. The amended document now includes specifics on the processes used to obtain consensus in the "Study Selection" subsection of the Methodology section, page number 6, lines 124–127. Again, thank you for your insightful comments.*

3. Line 125 how unpublished articles could be incorporated in a systematic review? It will reduce the reliability of the systematic review.

***Response:*** *Thank you for taking the time to review and provide insightful comments regarding the unpublished articles we included in our manuscript. We recognize that unpublished research plays a significant role in offering insightful information and increasing the sample size and number of studies included in the analysis, which can improve the statistical power and precision of the findings. We have carefully examined the inclusion criteria for unpublished articles in order to allay these worries. We provide a thorough explanation of the procedures followed to evaluate the accuracy and consistency of data obtained from unpublished sources, including the application of the Newcastle-Ottawa Quality Assessment Scale. We appreciate you bringing these crucial points to our attention once more. We value your feedback, and we hope that this will provide a clear explanation and a detailed description regarding the inclusion of unpublished articles.*

4. The exclusion criteria should be more specific regarding the caregivers, timeframe, only government facility or private NICU facility, study type (case report, case series, review papers etc.). This factors should be described in a well written manner.

***Response:*** *We appreciate your thorough review of our manuscript. We value your attention to detail and your recommendations for making our exclusion criteria more understandable. We would like to make it clear that the variables you brought up—caregivers (line 133), timeframe (line 140), facility type (line 137), and study type (line 142-143)—are considered in the updated manuscript's inclusion and exclusion criteria (page number 6-7, lines 131-143). We believe that these changes will allay your concerns without changing the essential components of our inclusion and exclusion criteria. Again, thank you for your insightful comments.*

5. The PICO framework or CoCoPop format should be followed.

***Response:*** *We appreciate your valuable feedback. Our revised manuscript now clearly states the PICO framework (page number 6-7, lines 133–136). The study's population, intervention, comparison, and outcome have all been precisely defined. We are grateful for your suggestions on improving the clarity of our study. Again, we thank you for your helpful comments.*

6. Line 144-145, discrepancies was resolved by TG and GGG, but was it in full text screening phase or initial title-abstract screening phase? Which software was used to screen the initial phase should be mentioned.

***Response:*** *We appreciate you taking the time to carefully review our manuscript and bringing out this oversight. We apologize for the confusion on lines 144–145. To be clear, two reviewers reconciled the differences at the full-text screening stage (page number 6, lines 125–127). We also recognize that the software utilized during the first title/abstract screening stage needs to be specified. To complete this review phase, we used Endnote X8. For clarification, this material has been included in the amended paper (page number 6, lines 122–123). Again, thank you for your insightful comments.*

Results:
1. The result section should add some demographic information of the parents. Preferably a Table.

***Response:*** We appreciate your insightful comments on our work. We are grateful that you suggested adding the parents' demographic information to the results section. While we understand the importance of demographic information in providing a comprehensive overview of the study population, it's important to note that our study is a systematic review and meta-analysis that focuses primarily on synthesizing findings from multiple studies. In our analysis, we included 11 articles with a total of 3,248 participants, so adding demographic information to the included 11 articles would be difficult and take these study designs away from their original intent. Instead, a summary table of the study characteristics of the included studies was included (page numbers 11-12, Table 1). We believe this method effectively addresses your concern. Once again, we appreciate your insightful remarks.

2. Line 288-289 should mention the exact AOR regardless three times.

***Response:*** *We appreciate you taking the time to carefully review our study. We have made the necessary revisions to lines 288–289 to provide the precise AOR values in our revised manuscript (page number 15, lines 299–300). Again, thank you for your valuable comments.*

Discussion:
1. The discussion should contain comparison with other region also regardless Nepal with representing the data.

***Response:*** *We thank you for your insightful comments and for taking the time to review our work. We would like to make it clear that a comparison of our results with those from other places outside of Nepal is already included in the discussion section. Relevant research from Rwanda, Kenya, Sri Lanka, Iran, Norway, India, Greece, Kenya, California, Vietnam, India, and London has been included to give a thorough overview of the topic and to emphasize the points of similarities and differences between our results and those reported in the literature (page number 15-17, lines 310-312, 323, 334, 344-345). Once again, we appreciate your valuable feedback.*

2. The compassion and sub-group analysis should distinguish showing the OR. How one group is more satisfied with another with the respective OR. Ref (Parental satisfaction with neonatal intensive care unit services and associated factors in Ethiopia: systematic review and meta-analysis. DOI: <https://doi.org/10.1186/s12912-024-01902-3>).

***Response:*** *We are grateful for your comments regarding our manuscript. We understand your interest in comparing satisfaction levels in the compassion and sub-group analysis using odds ratios (OR). To clarify, rather than being different, the overall OR of the sub-group analysis should be the same as compassion (parental satisfaction). Parental satisfaction and the subtotal OR for each group in the subgroup analysis are not the same, though (figures 4 and 5). Although we are grateful for the reference you provided, it's important to consider the distinct characteristics of our studies, such as the fact that we included a large number of studies with a large sample size compared to previous studies like the one you mentioned, which included a small number of articles with a smaller sample size. Thank you for bringing this to our attention, and we value your contribution to improving the clarity of our study.*

3. Line 345-346, this line is contradictory. I think, this similar type of review was done in the previously mentioned DOI. I have conflict of interest with this line.

***Response:*** *We appreciate the valuable reference you provided in the previously mentioned DOI. The sentences (Lines 345–346) under the Strengths and Limitations section have been omitted. You are absolutely right that our study is not the first systematic review and meta-analysis on parental satisfaction with care given at the NICU and its associated factors in Ethiopia. However, our study expands upon existing knowledge by providing a more comprehensive analysis.* *We included a large number of studies with a large sample size compared to previous studies like the one you mentioned, which included a small number of articles with a smaller sample size.*

4. Regardless of Africa only choosing Ethiopia is one of the greatest limitation of the study I think.

***Response:*** *We are grateful for your insightful comments. We agree that one important limitation of our study is that it only focuses on Ethiopia. We have specifically mentioned this restriction in the manuscript's limitation section (page number 18, line 359-360). We have suggested carrying out comparable studies in African nations to solve this constraint and direct future study, offering a more thorough grasp of the subject throughout the continent (page number 18, line 360). We believe that we have improved the overall quality of our study by openly addressing this limitation and suggesting potential future research areas. Again, we appreciate your valuable feedback.*

5. How associated factors contribute to this study should precisely mention in the conclusion section also.

***Response:*** *We really appreciate your insightful comments on our manuscript. We have updated the conclusion section to more clearly describe how associated factors affect this study in accordance with your suggestion. The association between these variables and parents' satisfaction with the services provided by the neonatal intensive care unit is now more succinctly summarized in the amended manuscript (page number 18, lines 366–374). We believe these modifications will improve the conclusion's overall impact and clarity.*

6. Authors contribution section should mention detailing the screening and publication bias section aslo.

***Response:*** *We thank you for your valuable comment regarding the author contribution section. As per your suggestion, we have corrected the revised manuscript (page number 20, lines 402–404). Again, thank you so much for your insightful feedback.*

Reference:
1. Ref 3, 30, 36, 38, 40, 45 should be rechecked and followed appropriate guideline.

***Response:*** *We are very grateful for your insightful comments. Based on your recommendation, we have carefully checked references 3, 30, 36, 38, 40, and 45. We have checked and corrected each reference in the updated manuscript. We believe that the amended manuscript now accurately and consistently cites the relevant literature.*

Others:
1. Fig 1 should mention PRISMA, variation of study means (location)?

***Response:*** *We appreciate your valuable feedback. Thank you for pointing out the methodology used in Figure 1. In order to make it clear that the PRISMA 2020 guidelines were followed during the study selection process, we have updated Figure 1 in our revised manuscript. Furthermore, we have updated the image to indicate that studies that had population differences are the subject of the "variation of study means (location)," which was an error. We believe these changes improve the figure's readability and comprehensiveness. Again, thank you so much for your valuable comment.*

2. Fig 2 was not exact manner, the remarketing line should be middle of the study and ES. Same for Fig 4 and 5.

***Response:*** *We appreciate your valuable comment regarding Figures 2, 4, and 5. We understand that there may have been some confusion about the figure elements. The following is represented by the lines in the figures:*

- *The red dot line represents the estimated pooled average prevalence across all studies, and this is intentionally placed at the midpoint of the study period to accurately represent the central tendency of the data.*
- *The black line indicates the boundary line that shows the prevalence can’t go beyond 0, because it cannot be negative.*

*Once again, we are very grateful for your review and the opportunity to explain the figure elements. We are willing to consider any particular recommendations that you may have for enhancing the data's visual representation.*

**Reviewer #4:** The paper is well written and the analysis is up to the standard. The PRISMA checklist is used and attached as per guideline of reporting a systematic review article. The discussion section is well argued and reference list is adequate. Congratulations team of authors

***Response:*** *We sincerely appreciate your encouraging and positive feedback. We are delighted that you found our paper well-written, the analysis up to standard, and the discussion well-argued. We value the recognition of our compliance with PRISMA guidelines and the adequacy of the reference list. We are very grateful for your encouraging remarks, which inspire us to keep up our research. Again, we want to thank you for your invaluable time and expertise.*
