# Supplementary material for: Parental satisfaction towards care given at neonatal intensive care unit in Ethiopia: A systematic review and meta-analysis
Source: PLoS One. 2024 Dec 5;19(12):e0313451. doi: 10.1371/journal.pone.0313451 (PMC11620403; doi:10.1371/journal.pone.0313451)
Supplement: Attachment — Submitted filename: Response to Reviewers.docx [file pone.0313451.s009.docx]

**Date: September 13, 2024**

**Subject:** Response to reviewer for the revised manuscript submission.

**Title:** Parental satisfaction towards care given at neonatal intensive care unit in Ethiopia: a systematic review and meta-analysis; [PONE-D-24-18838]

**Dear Editor and Reviewers,**

We appreciate your time and expertise in reviewing our manuscript. your valuable input has undoubtedly contributed to the enhancement of our manuscript. We appreciate your time and effort in reviewing the previous version and considering our responses to your comments. We are glad that the revisions we made in response to your previous comments have addressed your concerns and met your expectations. Once again, we sincerely appreciate your positive response and we are grateful for your thorough evaluation of our manuscript.

Thank you so much for your time and consideration.

Sincerely,

The authors

**Reviewer #2:** Review Report

Abstract: Needs further enrichment

**Response:** *We are grateful for your valuable feedback for our abstract to make enrich. We understand that the abstract could be more detailed/enrich. However, we are unable to make enrich/expand the abstract section because of the journal's strict word limit (no more than 300 words) unless we intend to avoid some sentences. Thank you once again for your valuable suggestions.*

Background: Relatively weak

**Response:** *We appreciate your thoughtful feedback. We have made some modifications in the background section based on your suggestion by adding one paragraph that is detailed about the neonatal intensive care unit and preterm newborn (page number 3, lines 59-67). We believe the details provided are adequate to illustrate the background and purpose of the research. If you have any further comments, suggestions, or specific areas you would like us to add/focus on, please let us know. Thank you once again for your insightful comment.*

Methods: Not comprehensive

**Response:** *We appreciate your review and valuable feedback on our methods section.* We worked hard to create a comprehensive methods section that provides a thorough overview of our manuscript. *If you have any further comments, suggestions, or specific areas you would like us to focus on, please let us know. Again, we appreciate your insightful comment.*

Result and discussion: Is weakly logical and the explanation should be rich with appropriate references.

**Response:** *We believe that the logic connecting our results to our discussion is sound and well-supported. Additionally, the references cited within the results and discussion are also appropriate. Please do not hesitate to point out any particular examples, where you believe the logic is weak or where you believe more references are needed. We appreciate for you valuable input.*
